# The Shape of Stability: Processing History and Functional Response in Thermosets, with a Focus on Filled Epoxies

**DOI:** 10.3390/polym18212704

**Published:** 2026-11-01

**Authors:** Andrea Szpecht

**Affiliations:** Poznan Science and Technology Park, Adam Mickiewicz University Foundation, St. Rubiez 46, 61-612 Poznan, Poland; andrea.szpecht@ppnt.poznan.pl

**Keywords:** epoxy composites, processing history, curing, filler reorganization, shape retention, rheology, electrical conductivity, multiple correspondence analysis

## Abstract

Assessing stability in filled epoxies requires specifying the intended material state and processing history. This structured narrative review examines local structural stabilization, physical support during fabrication, and functional retention, drawing on a broader corpus of 63 publications on filled thermosets. Multiple correspondence analysis (MCA) organizes evidence profiles from 60 empirical publications with complete codes by mapping co-occurring processing comparisons, structural observations, stabilization indicators, and functional-assessment stages. Carbon nanotube and clay studies show that structural plateaus indicate limited change in measured descriptors within observation windows, rather than global filler arrest or preservation of initial dispersion. Matrix cure markers cannot substitute for direct structural observations. In reduced graphene oxide composites, faster local network development observed optically does not necessarily correspond to higher conductivity in separately cured bulk specimens. In silica-filled epoxies, thermal programs must balance resistance to deformation against cure advancement; rheological evidence alone does not establish shape retention during printing. Electrical advantages established after one processing program can diminish after further heating, while recovery on cooling differs from retaining performance at elevated temperature. Meaningful stability comparisons therefore require observables, comparators, and readout conditions appropriate to the intended material state and processing sequence; transfer to other histories requires separate assessment.

## 1. Introduction

Processing a filled epoxy involves developing both a polymer network and a filler arrangement capable of providing the intended properties. Mixing establishes an initial dispersion, but the structure can continue to evolve as temperature and viscosity change and curing proceeds. In epoxy–clay systems, for example, preparation and the competition between structural development and gelation influence the resulting morphology [1]. In graphene-filled epoxies, mixing history can affect the spatial development of conductive paths and the bulk electrical response even at the same nominal composition [2]. For epoxy inks, resistance to deformation during curing is also needed to preserve the deposited shape. However, heating can initially reduce this resistance, whereas curing at a lower temperature may limit conversion through vitrification [3]. Processing conditions must therefore be considered alongside matrix chemistry, filler type and loading.

Here, stability means persistence of a specified material state or response over a stated observation period or processing history. Structural stabilization is assessed through limited further change in a defined structural descriptor, within the resolution and observation window of the measurement [4]. Preserving the initial dispersion and allowing conductive connectivity to develop can require different structural outcomes [2,4]. Physical support is the capacity to resist deformation under the loads encountered during fabrication; shape retention is the observed preservation of the object’s geometry through the tested process. Rheological evidence of support does not by itself demonstrate shape retention. Functional retention concerns whether the required material response is maintained under specified readout conditions and through the relevant further history. These assessments may overlap, but one does not establish another: recovery of an electrical response on cooling, for example, does not demonstrate stability while hot [5].

Previous reviews have examined how processing and curing influence structure and properties in epoxy systems. For epoxy–clay nanocomposites, this includes the effects of preparation, curing chemistry and competing routes of clay structure development on material properties [1]. Surendran et al. [6] review viscoelastic phase separation and domain development in epoxy blends, including the roles of gelation, vitrification and subsequent treatment. Although these domains differ from the added filler structures considered here, the review highlights the importance of relating morphological evolution to changes in the matrix. Reviews of cure kinetics also discuss both the effects of polymeric inclusions and nanofillers on the reaction and the scope and limitations of the kinetic models used to describe it [7]. A more recent review of graphene-filled epoxies covers dispersion, processing strategies, properties and characterization, including the limitations of using laboratory measurements to assess a finished component [8]. These reviews show how structural development depends on curing and processing, while also discussing what the available characterization methods can establish.

Previous reviews have also linked material behavior to fabrication and subsequent performance. For direct ink writing, Rau et al. [9] review how rheological measurements relate to extrusion, solidification and layer support. They also highlight the difficulty of reproducing the deformation and temperature histories experienced during printing in rheological tests. Reviews of particle-based multiphase inks consider rheology, printability and functionality together [10]. For conductive adhesives, Cui et al. [11] discuss how silver fillers, matrix and additive chemistry, curing conditions and subsequent heat treatment influence performance. Taken together, these reviews establish the importance of structural development, fabrication conditions and functional performance. Comparing their implications for a stability claim requires connecting the measured observable to the intended material state and examining whether the conclusion remains applicable at another processing stage or under different measurement conditions.

Addressing this problem requires specifying what is being compared and at which stage of processing the assessment is made. Comparing preparation routes at fixed nominal composition serves a different purpose from comparing formulations subjected to the same processing program. Formulation comparisons must also account for matrix modifiers: the graphene- and halloysite-containing epoxy nanocomposites studied by Lapčík et al. contained 10 wt% carboxyl-terminated butadiene–acrylonitrile rubber (CTBN), whereas the virgin epoxy reference did not [12]. The outcome also depends on the property selected: in copper-filled epoxies, Mutlu et al. reported lower tensile strengths than for the unfilled reference, although some formulations had higher flexural moduli [13]. Beyond formulation and property selection, the stage of assessment can affect the practical interpretation of the results. In the carbon-nanotube-filled pastes studied by Tsujimura and Inoue [14], the electrical advantage associated with a modified binder after a mild cure becomes much smaller after additional heating. In silver-filled epoxy composites, the relative ordering of reported resistivities for different processing histories within a formulation can also depend on the stage of assessment [15]. These examples show why selecting a processing program requires considering how further processing may affect an apparent benefit. A benefit observed for one manufacturing route may therefore require a separate assessment before it can be expected under another.

Relating results from different measurements requires attention to what each method measures and under which conditions. Structural imaging, rheology, cure markers and functional tests probe different aspects of a material, sometimes using different specimens or geometries. Kernin et al. [2] combine optical observations in a droplet with electrical measurements on separately cured bulk specimens. Their results associate preparation-dependent differences in morphology with differences in electrical response, but do not establish a quantitative link between local morphology and bulk transport. Comparing such findings can distinguish the differences demonstrated experimentally from the evidence still needed to explain how they developed. Studies comparing final properties do not necessarily track the full evolution of the structure, and studies of matrix cure do not necessarily measure functional performance. Whether these results can be interpreted together therefore depends on the question being asked, the specimens examined and the comparability of measurement conditions, rather than simply on the number of techniques used.

This structured narrative review therefore asks: what remains stable in a filled epoxy system, and what evidence supports extending that conclusion to another processing stage or material response? The detailed material synthesis focuses on filled epoxy systems. The broader, deliberately selected corpus of filled-thermoset studies provides the context for the publication-level evidence profiles, with uncured materials and controls retained where they help interpret processing stages and material comparisons. Systems with carbon-based fillers or layered silicates, silica-containing inks and silver-containing systems provide selected comparisons of structural evolution, physical support and electrical behavior. Three subsidiary questions guide the analysis. What do structural plateaus and matrix cure markers establish about filler stabilization? How does rheological evidence of support relate to shape retention during curing? How does further heating affect the interpretation of an electrical result or an apparent advantage of one formulation over another? Each comparison is assessed in terms of the measured observable, reference state, observation window, and comparability of formulation, specimen geometry and processing or readout conditions. These criteria are described in Section 2.3 and Section 2.4 and applied in Section 4, Section 5 and Section 6, with observations distinguished from mechanistic interpretations and gaps in the reported conditions explicitly identified.

Multiple correspondence analysis (MCA) supports this review by organizing publication-level profiles that describe the processing histories compared, the structural evidence reported, the basis for assessing stabilization and the stage of functional assessment. It highlights recurring combinations and contrasts, helping distinguish evidence about the resulting materials from evidence about how they developed. The comparability of measurement conditions is assessed separately, because a single publication may report observations from different specimens and processing programs. These profiles describe the evidence available in the selected studies; they do not rank study quality or establish mechanisms or causality. Their role is to show which questions the reported measurements can address and to guide the detailed comparison of material systems.

The contribution of this review is a comparative synthesis of how the observable, reference state and subsequent processing history change the meaning of a stability claim in selected filled epoxy systems. Combining material comparisons with publication-level evidence profiles helps distinguish a demonstrated outcome from the additional evidence needed to explain its development or extend it to another stage. The synthesis supports choosing what to measure, which reference state to use and which processing stages require assessment. This guidance is qualitative and limited to the systems and conditions examined. Figure 1 summarizes the questions guiding these comparisons.

## 2. Review Scope, Evidence Base, and Methods

### 2.1. Review Design and Scope

This structured narrative review combines comparisons of experimental results with multiple correspondence analysis of publication-level evidence profiles. The detailed synthesis focuses on filled epoxy systems selected from a broader set of studies on filled thermosets. It examines structural plateaus and cure markers, physical support during fabrication, and electrical response through cure and further heating.

Eligible systems included filled thermosetting matrices, predominantly epoxy, as well as resins without hardener, inks and pastes used as controls or materials at intermediate processing stages. The scope included fillers added to the matrix or generated in situ and extended from preparation and mixing through cure to further thermal or aging treatment. Relevant evidence included observations of structural change, physical support or processability, matrix markers, or functional response associated with an identified process. Studies did not need to examine all these aspects. Missing information about formulation or measurement conditions restricted the comparisons that could be made but did not automatically exclude a publication. Further eligibility details are provided in Supplementary, Section S1.1.

### 2.2. Literature Identification and Selection

Scopus was searched on 8–10 September 2026, and Web of Science Core Collection on 14 September 2026. Queries combined terms for thermosets and fillers with terms related to processing, cure, structural reorganization, stabilization, and measurement. Separate searches addressed primary studies and earlier reviews. Targeted web searches and limited citation tracking through the OpenAlex and Semantic Scholar APIs supplemented the database searches; some forward searches examined only a subset of citing publications.

The corpus was assembled through iterative, priority-based selection rather than inclusion of all publications meeting the broad topical criteria. Selected candidates underwent full-text assessment, and screening priorities differed between search routes. The 63-publication analytical corpus was assembled by 14 September 2026. Detailed material comparisons were chosen after extraction and coding; that choice did not determine corpus membership.

Worksheet 02-Search provides the 14 query strings, dates, search restrictions, and export counts. Worksheet 15-Selection records screening decisions, reasons for nonselection, and links to the coded publications. Supplementary, Sections S1.2 and S1.3 and Figure S3, describe the selection routes and their limits. Web of Science was searched without a publication-year limit (All years). Counts are reported by route because the searches and supplementary checks overlap.

### 2.3. Evidence Corpus, Extraction and Comparative Synthesis

The evidence base contains 63 publications: 55 empirical studies, six empirical publications assigned a control role and two modeling studies [16,17]. Of the 61 empirical publications, 60 enter the main MCA with equal weight. REV-049 remains in the review and evidence base, but its L code is unresolved [18]. It is therefore excluded from defining the main MCA axes. Two sensitivity scenarios include 61 publications, with an assumed LE or LM code for REV-049; neither assignment is treated as confirmed. The two models remain outside MCA. Bibliographic roles and membership are recorded in SI, worksheet 03-Publications. Publications need not represent independent experiments; REV-033 and REV-048 partly share data [3,19].

Data extracted from the full texts included material composition and geometry, processing conditions, structural and functional measurements, stabilization markers, comparators and source locations. Selected details were checked against the source publications before the records in SI, worksheet 04-Evidence, were finalized. The extraction distinguished measured observations from the source authors’ mechanistic interpretations and the conclusions supported in this review.

For the material synthesis, each result was considered together with its comparator and measurement conditions. Comparisons were first examined within individual publications and then considered across material systems, taking account of what was observed, over which time window and relative to which reference state. Proximity on an MCA map was not taken to imply comparable experimental conditions. Measurement uncertainty, missing conditions and unresolved inconsistencies in the sources were explicitly noted. No pooled effect, common retention percentage or ranking across filler systems was calculated. The analytical units, extraction procedures and handling of source limitations are described in Supplementary, Sections S2 and S3. Evidence strength is assessed at the level of each material claim, using the directness and scope of the measurement, replication and uncertainty where reported, model dependence, and transfer between specimens or conditions. An unreported quantity remains unreported. No common quality score or low/medium/high ranking was imposed: equal publication weights describe the distribution of evidence profiles, not equal confidence in the underlying material claims.

### 2.4. Evidence Profiles and Condition Linkage

Each publication was assigned one category for each of four categorical variables. H indicates whether processing histories were deliberately compared at the same nominal formulation; S describes the structural evidence; L identifies the basis for stabilization; and F records whether and at which stage a separate function was assessed. Table 1 defines the 13 categories used in the analysis.

When more than one category applied, the code was selected according to the priorities LS > LP > LM > LE and FR > FE > FP. These priorities determine which category represents a publication; they do not rank evidence strength or study quality. Category frequencies therefore do not capture all measurements reported in each publication.

The unit analyzed by MCA is the publication-level profile, which may combine observations from different specimens or conditions. Separate structure–stabilization (S–L) and structure–function (S–F) qualifiers describe how closely the conditions of the relevant observations match. They distinguish locally matched evidence, partial linkage, known mismatch, unresolved linkage, a confirmed gap in the protocol description, and the absence of an adequate pair of observations. These qualifiers do not establish complete processing–structure–function chains. Full definitions are provided in SI, worksheet 05-Codebook. Assigned codes, measurement-condition qualifiers and the rationale for each assigned code are provided in Supplementary, worksheet 06-Coding. The qualifiers and limits of the coding scheme are explained in Supplementary, Section S4 and Figure S2.

LM requires a traceable matrix state or transition serving as a stabilization reference, not merely a reported T_g_ or other property. Measured, modeled, indirect and transferred markers remain admissible, including documented coder interpretation. Neither direct filler arrest nor a common specimen for every measurement is required. LE requires its own operational basis; absence of LM does not automatically imply LE or L0. The definitions and their interpretive limits are documented in Supplementary, Section S4.1, and worksheet 05-Codebook.

### 2.5. MCA and Sensitivity Analysis

MCA describes how H/S/L/F categories occur together across publications [20]. The main analysis uses a complete 60 × 13 indicator matrix, with H, S, L and F as the only active variables and equal publication weights. Singular value decomposition of the standardized indicator matrix yields nine nonzero dimensions. Material, bibliographic and condition descriptors are supplementary and do not determine the axes. Their associations are descriptive, without significance testing.

Raw, uncorrected inertia describes the dispersion of the coded profiles. Category contributions show which categories contribute most to an axis, while cos^2^ indicates how much of a point’s squared distance from the origin is represented by a dimension or plane. These measures do not indicate experimental quality or physical effect size. Publications with identical code profiles have identical coordinates by construction; this does not establish validated clusters. Matrix standardization, diagnostic measures and the locations of numerical outputs are documented in Supplementary, Section S5.1. Alongside raw inertia, SI reports Benzécri-adjusted values and percentages using Greenacre’s denominator [21] (Supplementary, Section S5.1). These corrections summarize inertia without rescaling the map coordinates, contributions or cos^2^. The 1/4 eigenvalue criterion is a heuristic, not a significance test or proof that exactly four dimensions are correct. Axes 1–4 are displayed for their interpretable contrasts and representation of particular profiles; all nine are retained, with 1–5 remaining the diagnostic space.

Exploratory sensitivity analyses assign H0 to REV-001, remove the six controls, merge FP with FE, or omit each of the 60 publications in turn. Common publications are compared in axes 1–2, 3–4 and 1–5 using distance-rank correlation, normalized Procrustes similarity [22] and the minimum principal-angle cosine [23]. The 63 perturbation solutions are each compared with the main solution in the three spaces, giving 189 comparisons. The main solution and two REV-049 scenarios form three pairs, giving nine further comparisons across the same spaces. The resulting 198 comparisons each have three descriptive similarity measures. Supplementary reports leave-one-out medians, quartiles and minima separately from other perturbations and scenarios. The scenarios were specified before their calculation, but after the initial analysis and the coding uncertainty were known; the axes-3–4 checks followed analysis of the 1–2 and 1–5 spaces. These controls are exploratory, not preregistered or confirmatory.

A full analysis restricted to conditions matched to the functional specimens was not performed because the extracted information was insufficient to apply alternative coding consistently across the corpus. Some associations may also arise partly from the code definitions, particularly the link between structural observation and local stabilization. MCA describes the evidence available in the selected publications; it does not support population estimates or establish causality or shared mechanisms. Results are presented in Section 3.

No axis-significance *p*-values were calculated. Unrestricted permutations would test independence of fields while potentially creating combinations incompatible with their definitions, such as LS without the required structural evidence. A valid restricted null model and exchangeability assumptions were not specified. A publication bootstrap would address composition under a resampling model, not independently validate coding or shared-data independence. The reported sensitivity coefficients are descriptive and are not inputs to a Benjamini–Hochberg correction.

### 2.6. Conduct, Tools and Data Availability

The author conducted searching, screening, extraction and initial coding with AI assistance, without an independent second coder. A subsequent source-based author reassessment was compared with the initial codes before differences were resolved. Sixteen of the 61 empirical publications were sampled without replacement. One became a training case and was excluded from comparison without drawing a replacement, leaving 15 randomly selected publications and two additional targeted cases. All 68 first reassessment assignments were completed without interpretive AI assistance. Later resolution of differences involved AI-assisted examination and final decisions by the author. The LM/LE instruction was clarified between coding stages, before further author reassessment, so agreement does not measure repeat coding under identical rules. Unweighted Cohen’s κ and nominal Krippendorff’s α summarize agreement for these 15 publications and, separately, for the combined set of 17 (Supplementary, Table S4). Supplementary, Section S4.3, and worksheet 13-Reassessment describe the selection, human and AI roles, and agreement calculated before resolution of differences.

The numerical implementation uses Python (version 3.12.14)/NumPy (version 2.3.5). Supplementary_Code.zip supplies the final evidence base, configuration, code for fitting indicator-matrix MCA, complete numerical results, profile membership and figure-data generators. The workbook contains static results and does not itself recalculate MCA. Supplementary, Section S5.3, and the package README describe reproduction and checks. Numerical reproduction does not validate source interpretation or coding. The author takes responsibility for the content, including AI-assisted analyses and drafting.

## 3. Evidence Profiles Across the Reviewed Studies

### 3.1. Evidence Profiles and Their Interpretation

Which combinations of processing comparisons, structural observations, stabilization criteria and functional measurements occur together in the reviewed publications, and how can they inform the material comparisons? MCA addresses this question by describing publication-level evidence profiles. A profile summarizes the types of evidence reported in a publication, whereas a conclusion about a material concerns a specific experimental result, its comparator and its conditions. Keeping these levels separate helps clarify whether a comparison is supported by an observed structural trajectory, an endpoint, evidence of physical support or a functional measurement after further processing. It also avoids treating different types of measurements as directly comparable material properties.

The 63-publication evidence base contains 61 empirical publications and two models. The main MCA includes 60 empirical publications with complete H/S/L/F codes: 54 studies and six controls. REV-049 remains in the evidence base with unresolved L. The 13 active categories form 25 distinct combinations: 13 profiles shared by two or more publications and 12 single-publication profiles. This number is obtained by counting exact four-code combinations, not by a clustering algorithm. Each publication has equal mass; a shared profile therefore carries the combined mass of its members.

In Figure 2, circle area is proportional to the number of publications sharing a profile. P1–P13 identify shared profiles in the accompanying key, while profiles represented by a single publication carry author–year labels. Identical codes give identical coordinates throughout the MCA space. Nearby points with different profiles, however, are similar within the displayed projection and may differ along other dimensions. Color indicates the summed cos^2^ for the displayed plane: the fraction of a point’s squared distance from the full-space centroid represented by those axes. It shows how well the plane represents the profile, not the quality of the study. Profiles retain the same identifiers and circle areas on the second map, although their colors may change because their representation differs between projections. Profile labels follow the lexicographic order of current code combinations. Worksheet 11-Profiles supplies profile frequencies and the mapping from REV identifiers to profiles.

The category frequencies reveal a distinction relevant to the material synthesis. Deliberate comparisons of histories at the same nominal formulation were coded in 44 publications, whereas displayed structural trajectories were recorded in 16. Structural states or variants without a decisive trajectory were recorded in 32 publications. History comparisons were therefore more common than displayed structural trajectories. This distinction matters because comparing the results of different processing histories does not, by itself, show how the structure developed between the measured states. These frequencies reflect the selected corpus and the coding priorities; they do not describe the wider literature or count every measurement reported in each publication. For example, publications assigned a later functional-assessment stage may also report functional measurements during processing, and a local structural stabilization code can take priority over an accompanying matrix marker. In the main 60-publication analysis, L comprises 14 LE, 28 LM, 10 LP and eight LS assignments.

The publication key for both MCA maps is included here. P1–P13 denote shared exact profiles, not clusters or quality grades. Singleton and shared membership below refer to the main 60-publication analysis; the complete frequency table and REV-to-profile mapping are in SI, worksheet 11-Profiles.

Single-publication profiles: Trigg 2021 [24]; Leonardi 2026 [25]; Chapartegui 2012 [26]; Chen 2003 [27]; Kim 2001 [28]; Pekol 2024 [29]; Fukumoto 2022 [5]; Meng 2005 [30]; Albertini 2026 [31]; Ilyina 2026 [32]; Kong 2003 [33]; Gao 2012 [34].

P1 (n = 3; H0/S0/LP/F0): Chiou 2001 [35]; Romberg 2025 [19]; Nohales 2013 [36].

P2 (n = 2; H0/S1/LE/FE): Sumfleth 2011 [37]; Wang 2014 [38].

P3 (n = 5; H0/S1/LM/F0): Furuta 2026 [39]; Sanctuary 2009 [40]; Lagasse 2002 [41]; Fukushima 1978 [42]; Tiwari 2023 [43].

P4 (n = 2; H0/S1/LM/FE): Jie 2024 [44]; Liu 2019 [45].

P5 (n = 4; H1/S0/LM/F0): Harsch 2008 [46]; Kortaberria 2006 [47]; Surendran 2021 [48]; Altmann 2003 [49].

P6 (n = 2; H1/S0/LP/F0): Weißer 2023 [50]; Romberg 2023 [3].

P7 (n = 7; H1/S1/LE/FE): Zhu 2009 [51]; Nagarajan 2021 [52]; Hu 2008 [53]; Li 2026 [54]; Liu 2026 [55]; Kim 2020 [56]; Wu 2017 [57].

P8 (n = 7; H1/S1/LM/FE): Dean 2005 [58]; Wu 2019 [59]; Sakaniwa 2016 [60]; Zhao 2008 [61]; Morais 2019 [62]; Nuhiji 2013 [63]; Zhou 2008 [64].

P9 (n = 3; H1/S1/LM/FR): Tsujimura 2022 [14]; Rivers 2018 [15]; Rivers 2017 [65].

P10 (n = 4; H1/S2/LE/FE): Han 2021 [66]; Yakovenko 2017 [67]; Chen 2026 [68]; Mao 2024 [69].

P11 (n = 3; H1/S2/LM/FE): Martin 2005 [70]; Rýznarová 2008 [71]; Wilson 2005 [72].

P12 (n = 3; H1/S2/LS/F0): Yourdkhani 2015 [4]; Dell’Erba 2017 [73]; Hernandez 2007 [74].

P13 (n = 3; H1/S2/LS/FE): Prasse 2003 [75]; Belijar 2016 [76]; Kernin 2019 [2].

### 3.2. Main Contrasts in the First Two Dimensions

Dimensions 1 and 2 account for 23.78% and 18.42% of raw inertia, together 42.20% (Figure 2). With Benzécri-adjusted values and Greenacre’s denominator, their combined share is 76.64%; the two percentages use different definitions. Neither is a physical effect size or a stand-alone measure of map stability. Figure 3 shows category contributions; signed principal coordinates are supplied in Supplementary, worksheet 08-MCA-categories. Publication points represent full profiles, not individual categories.

On Dimension 1, proxy-based structural evidence (S0), the absence of a separately assessed function (F0), and physical-network response or support (LP) lie on the positive side. They contribute 20.9%, 15.4% and 11.4% to the axis, respectively. Structural trajectories (S2) and function assessed after a manufacturing program (FE) lie on the negative side, contributing 10.8% and 9.3%. This pattern reflects how categories occur together across publication profiles; it does not mean that structural observation necessarily accompanies a functional measurement. Profile P12 illustrates this distinction. It combines history comparisons, structural trajectories and local stabilization with no separately assessed function (H1/S2/LS/F0). Shared by Yourdkhani and Hubert [4], Dell’Erba et al. [73] and Hernandez et al. [74], it lies on the negative side of Dimension 1 despite its F0 assignment. The practical distinction is between the types of evidence used to support a material conclusion. For example, a rheological response may appropriately address a fabrication question even when the selected filler architecture is not directly imaged.

Dimension 2 more clearly separates structural trajectories and local stabilization from structural states and operational endpoints. The S and L fields together contribute 86.2% to this axis. Local limitation of structural change (LS) and structural trajectories (S2) have positive coordinates, whereas structural states (S1) and operational programs or thresholds (LE) have negative coordinates. LS makes the largest category contribution, at 30.3%, while S1 and S2 each contribute approximately one-fifth (Figure 3). The axis therefore distinguishes the types of observations used to discuss stabilization. However, this contrast partly reflects the coding definitions: all eight LS assignments in the corpus occur with S2. Identifying a local limitation of change requires suitable structural evidence, so this association cannot be interpreted as an independently discovered physical coupling.

The two axes distinguish how evidence is combined, rather than successive levels of experimental completeness. A field’s contribution also does not measure its importance to the material question. The history field H contributes only 2.3% to Dimension 2 but 29.1% to Dimension 5. A processing-history comparison can therefore be central to the material interpretation without dominating the first plane.

### 3.3. Later Dimensions and Profile Representation

The first plane represents some profiles poorly: 10 of the 60 publications have summed cos^2^ below 0.20. This threshold is a descriptive flag, not a significance criterion. A point near the center with low cos^2^ may differ substantially from other profiles along dimensions outside the displayed projection. on the map. Its position should therefore not be taken to mean that the publication provides little useful evidence.

Dimensions 3–4 provide an auxiliary projection of differences poorly represented in the first plane. They add 25.67% of raw inertia (13.18% and 12.49%), bringing the first four dimensions to 67.87%. Their combined share using Greenacre’s denominator is only 2.22%, indicating a weaker global contrast after correction. In the main solution, Dimension 3 contrasts positive FP and LM coordinates (28.36% and 20.50% contributions) with negative LP and LE coordinates (23.06% and 13.83%). LM can represent measured, modeled, indirect or transferred markers; it does not imply a common experimental criterion or a measured filler-arrest time.

Dimension 4 is primarily characterized by FR on the positive side (62.12% contribution), rather than by an equally balanced FR–FP contrast. H0 lies on the negative side and contributes 13.32%; FE contributes 6.69%. Together, axes 3–4 describe selected differences in stabilization basis and functional-assessment stage. They are not physical quantities or stages of increasing durability.

These contrasts can be seen on the publication map. Profile P9, shared by Tsujimura and Inoue [14] and Rivers et al. [15,65], combines history comparisons, structural states, matrix markers and functional assessment extending to a separate further history (H1/S1/LM/FR). It lies on the positive side of both dimensions in Figure 4. The two publications assigned FP—Kim [28], which reports mold-filling length as a measure of processability, and Leonardi et al. [25], which measures absorbance during processing—lie on the positive side of Dimension 3 but the negative side of Dimension 4. Their shared FP code therefore encompasses different types of functional measurements. In the latter study, the optical response also serves as a structural proxy, so the structural and functional evidence are not fully independent. Their positions indicate similarities in the coded evidence, without establishing equivalent functions or mechanisms. Because FP contains only two publications, its contribution to the later dimensions should be considered alongside the sensitivity results in Section 3.6.

Representation also needs to be distinguished at the category and publication levels. The FR category has summed cos^2^ of 0.037 on Dimensions 1–2 and 0.820 on Dimensions 3–4. These values describe how well the category is represented, not how well each of the six publications assigned FR is represented. For profile P9, the corresponding values are 0.113 and 0.843. The second map therefore shows this profile much more clearly, highlighting a combination of evidence central to the review’s comparisons of electrical response after further heating.

Figure 5 contrasts three situations. P13, represented here by Kernin et al. [2], is already well-represented on Dimensions 1–2, with cos^2^ of 0.855. P9, which includes Rivers et al. [15], gains most of its representation from Dimensions 3–4. P8, which includes Wu et al. [59], remains poorly represented across the first five dimensions, with cumulative cos^2^ of only 0.410. P8 combines history comparisons, structural states, matrix markers and function after a manufacturing program (H1/S1/LM/FE). Although the first five dimensions account for 78.46% of overall raw inertia, this does not guarantee good representation of every profile.

The bar segments show how much of each profile is represented, with colors identifying blocks of dimensions rather than values on the continuous cos^2^ scale used in the maps. Each bar gives the cumulative representation across Dimensions 1–5; the remainder up to one is the fraction of the profile’s squared distance represented by Dimensions 6–9. Figure 5 therefore shows why the overall inertia retained by a projection is insufficient to judge how well it represents a particular profile.

The second plane remains useful for displaying these specific profiles, but its interpretation is conditional on this corpus and coding. It is sensitive to individual publications, particularly REV-009, and to merging FP with FE; similarity of the plane as a whole does not guarantee preservation of the meaning of each axis (Section 3.6).

### 3.4. Recurring Evidence Profiles Across Material Systems

The two most frequent profiles, P7 and P8, each occur in seven publications. Both combine history comparisons, structural states and function after a manufacturing program (H1/S1/FE), but differ in stabilization basis: an operational reference in P7 and a matrix marker in P8. Their recurrence does not establish a common physical pathway or equal evidence strength.

P12 provides a different basis for comparison. Yourdkhani and Hubert [4], Dell’Erba et al. [73] and Hernandez et al. [74] report structural trajectories and evidence of local stabilization, without a separately assessed function in the selected profile. This profile groups studies of CNT dispersion, in situ formation of Ag particles and epoxy–clay blends, allowing their observation windows to be compared. However, optical observations of dispersion, scattering profiles and fitted descriptors provide different kinds of information, so the evidence for stabilization is not equivalent across these studies. In the epoxy–clay study, the authors report eventual stabilization, but the displayed profiles continue to evolve toward the recorded endpoints and do not demonstrate a sustained plateau. The shared LS code therefore does not establish a common arrest event or comparable temporal resolution.

Physical support provides another useful comparison. Pekol et al. [29] combine history comparisons, proxies for the selected architecture, physical support and a functional endpoint (H1/S0/LP/FE). Romberg and Kotula [3] belong to profile P6, which has the same H/S/L codes but no separately assessed function (H1/S0/LP/F0). Both studies report rheological evidence relevant to support without directly observing the selected filler architecture. The former demonstrates shape retention and fabrication; the latter develops a thermal program from rheology and conversion without testing whether a printed part retains its shape. Their different F codes distinguish the outcomes assessed, while both studies can inform processing decisions. Observations of liquid-crystalline domains in the Pekol study are not included in its silica-network profile.

The electrical studies in P9 illustrate why the same functional-stage code can cover different outcomes. Tsujimura and Inoue [14] report that an initial difference between formulations largely disappears after additional heating. Rivers et al. [15] show that cycle endpoints can shift in opposite directions for different combinations of formulation and processing program. Rivers et al. [65] report no detectable electrical change during a subsequent heating scan, without establishing exactly zero change or a quantitative retention value. FR indicates that function was examined beyond the initial preparation history; it does not establish improved performance or general durability. Similarly, FE does not establish that a measurement was made after cooling. The material state at the time of measurement remains essential to each comparison.

These examples show how the profiles identify recurring combinations of reported evidence and help select studies for closer comparison. Recurring profiles across different materials do not establish independence from filler chemistry. Filler group, included as a supplementary descriptor, was associated with Dimension 1 (η^2^ = 0.445). This value describes the proportion of variation in publication coordinates associated with differences between filler groups. This descriptive association is neither a significance test nor evidence of causality. It does, however, caution against assuming that materials with similar evidence profiles are otherwise comparable.

### 3.5. Measurement Conditions and Limits of Comparison

A well-represented profile may still combine measurements made under different conditions. P13 combines history comparisons, structural trajectories, local stabilization and function after a manufacturing program (H1/S2/LS/FE). The three publications sharing this profile occupy the same point on the MCA maps but differ in how their structural observations relate to the specimens used for functional measurements. In each study, the structural trajectory and evidence of local stabilization come from a locally matched observation series. This agreement does not automatically extend to the functional measurements.

Prasse et al. [75] observed nanofibers in neat hardener under conditions different from those used for the bulk epoxy–amine composite. Belijar et al. [76] used different filler contents and geometries for direct imaging and measurement of the quantitative functional endpoint. Both structure–function pairings are coded as known mismatches. In Kernin et al. [2], the optical droplet experiment and the separately cured bulk specimens share the nominal formulation and cure temperature, but differ in geometry and measurement schedule. This pairing is therefore coded as partially linked. The same H1/S2/LS/FE profile can thus cover different degrees of comparability between structural and functional measurements.

These distinctions are recorded separately from the MCA coordinates in Supplementary, Figure S2. Eight publications have a locally matched structure–stabilization series. For structure–function linkage, 22 publications have a partial link and 12 a known mismatch. Partial linkage may involve related conditions or companion specimens whose conditions have not been fully reconciled. A known mismatch limits the conclusions that can be drawn by combining the measurements, without invalidating either measurement. The remaining qualifiers and their full distribution are described in Supplementary, Section S4.2 and Figure S2. The absence of an adequate pair differs from a known mismatch, while unresolved linkage differs from a specific omission confirmed in the source. These pairwise assessments cannot be combined into a count of complete processing–structure–function chains.

Conclusions about a material therefore depend on the conditions of the individual measurements, even when its publication profile is well-represented on the maps. The qualifiers guide interpretation but do not replace an MCA restricted to conditions matched to the functional specimens. No such analysis was performed (Section 2.5).

### 3.6. Sensitivity and Scope of Interpretation

The first plane retains broad similarities under the examined perturbations (Supplementary, Figure S1). Distance-rank correlations are 0.994 for H0 assigned to REV-001, 0.980 when FP is merged with FE, and 0.963 without the six controls. Across 60 leave-one-out analyses the minimum is 0.985 (REV-043). The leave-one-out median Procrustes similarity is 0.999696. These summaries support a descriptive reading of the main contrasts in this selected corpus, not invariance under every change or generalization to the literature.

Later directions are more sensitive. In 5D, merging FP with FE yields a distance-rank correlation of 0.836 and minimum principal-angle cosine of 0.558; omitting REV-027 gives 0.976 and 0.216, respectively. For axes 3–4, the median Procrustes similarity across single-publication omissions is 0.999. Omitting REV-009 reduces it to 0.629, with a distance-rank correlation of 0.768 and a minimum subspace cosine of 0.217. Merging FP with FE moves the dominant FR contrast from axis 4 to axis 3. Conversely, both omissions from the partly shared-data pair REV-033/REV-048 give 3–4 Procrustes similarity near 0.994. The REV-049 LE and LM scenarios give 0.997 and 0.998 on the common 60 publications. These targeted results neither resolve REV-049 nor establish independence of all publications. Supplementary reports full results and separate leave-one-out quartiles.

Within this corpus, the first plane mainly distinguishes the types of structural evidence and the reported bases for stabilization. Dimensions 3–4 make differences in the stage of functional assessment more apparent. These contrasts help organize studies according to the questions their measurements can address. The material comparisons then examine the individual results, comparators and conditions underlying the profiles. The MCA maps do not establish validated mechanistic clusters, causality or a ranking of study quality. Profile frequencies describe a deliberately selected set of publications, and publication counts do not represent numbers of independent experimental replications. The following sections return to specific results and measurement conditions to examine local structural stabilization, physical support and electrical response as processing continues.

## 4. What Structural Plateaus and Cure Markers Establish

### 4.1. Structural Evolution and Local Stabilization

The meaning of structural stability depends on the feature observed during processing. The structural comparisons discussed here and summarized in Table 2 concern optically visible CNT agglomerates, clay structures sampled by scattering, and model-derived descriptors of Ag nanoparticles formed in situ. Each measurement can reveal changes in a particular feature within its resolution and observation window. Interpreting that result requires relating the observed feature to the preparation history and assessing whether later observations or an additional processing stage support persistence of the same state.

Yourdkhani and Hubert [4] provide a particularly informative control. In their Case A experiment, epoxy containing 0.1 wt% CNTs was heated from 70 to 120 °C at 2 °C min^−1^ and then held at 120 °C, with and without hardener. The optical dispersion index decreased during heating and subsequently reached a plateau in both systems. This plateau indicates limited further change in the index, rather than preservation of the starting dispersion. This conclusion applies to the agglomerates resolved and sampled optically; it does not establish immobilization of the entire CNT population.

Viscosity measurements performed separately under the corresponding temperature programs help distinguish the optical plateau from changes associated with curing. During the Case A hold, viscosity remained approximately constant without hardener but increased in the reacting formulation, although both optical indices plateaued. Under this program, hardener-mediated curing was not necessary for the optical plateau, and the plateau did not independently identify chemical gelation. Case B, which used the same 2 °C min^−1^ heating rate but reached 180 °C, shows why the role of curing must be considered for each thermal program. In the reacting formulation, the optical dispersion index stabilized during heating, which the authors associated with a pronounced viscosity increase as crosslinking progressed [4]. Together, these observations are consistent with curing contributing to restricted structural evolution, but an optical plateau alone does not establish when curing begins to restrict that evolution or identify a gel point.

Hernandez et al. [74] examined structural evolution at a different scale in epoxy/clay blends modified with poly(methyl methacrylate) (PMMA). Their sample designation S2 refers to preparation using a solvent and an ultrasonic probe, whereas S4 refers to a melt-mixing route. During cure at 135 °C, small-angle X-ray scattering (SAXS) profiles and model-derived tactoid-thickness distributions evolved over measurement periods extending to 30–36 min. The authors reported eventual profile stabilization, but the displayed endpoints do not establish a sustained plateau interval. The source also assigns the 30 and 36 min endpoints inconsistently to S2 and S4; Table 2 therefore reports the combined range without assigning either endpoint to a specific sample.

The measurements establish continued evolution of the clay structures represented within the scattering window. The fitted thickness distributions describe stacks of clay layers through a structural model, while large agglomerates may remain outside the accessible scattering-vector range. Neither the displayed evolution nor the reported final stabilization establishes complete exfoliation throughout the material. Hernandez et al. [74] also interpret continued SAXS evolution beyond the reported gel time of approximately 15 min as possible post-gel reorganization. However, the method used to determine that gel time is insufficiently described, so the timing of structural evolution relative to gelation cannot be independently established from the reported evidence.

Kong and Park [33] provide a complementary result from time-resolved small-angle X-ray diffraction (SAXD). In their epoxy–clay system cured with diaminodiphenyl sulfone (DDS), the gallery spacing increased in stages, with an interval of little change followed by renewed expansion. The continued measurements directly establish that the apparent plateau was transient. An observation ending within that interval would miss the later expansion. Cure conversion was assigned from separate differential scanning calorimetry (DSC) measurements, and chemical gelation was not independently located in the SAXD series.

These two clay studies follow different aspects of structural development. Kong and Park [33] track the spacing between clay layers, whereas Hernandez et al. [74] derive tactoid-thickness distributions from fitted scattering profiles. A plateau in layer spacing does not establish that the tactoid-thickness distribution is stable, and neither measurement captures all clay-containing structures. The systems also differ in matrix composition, including the presence of PMMA, preparation route, and cure conditions. These differences prevent the characteristic times from being treated as measurements of one common stabilization event. Because these factors vary together, the cross-study comparison does not isolate their contributions to the differing trajectories. The later observations support different conclusions: Kong and Park [33] demonstrate renewed expansion after a temporary plateau, whereas the displayed endpoints in Hernandez et al. [74] do not establish sustained late stability.

A separate processing stage can test another aspect of persistence. Dell’Erba et al. [73] studied Ag nanoparticles formed in an epoxy system under visible-light irradiation and reported nearly unchanged SAXS profiles beyond 420 s. A separate comparison before and after thermal post-cure at 150 °C for 1 h showed a small decrease in fitted radius of gyration and a Porod exponent closer to 4. The authors interpreted these changes as particle rounding without uncontrolled aggregation. Thus, additional processing altered selected particle descriptors without demonstrating a general loss of dispersion stability. The particle parameters are model-derived. The gel estimate uses a conversion of approximately 0.03, obtained from G′–G″ crossover and FTIR measurements in thermally cured DGEBA/BF_3_·MEA at 140 °C. This conversion was applied to the photochemical conversion curves. The transferred reference is therefore conversion, not gel time; gelation was not independently measured in the photochemical SAXS series.

The CNT, clay, and Ag examples illustrate distinct outcomes: an optical descriptor that stabilizes after an earlier dispersion change, a temporary pause followed by renewed layer expansion, reported stabilization without a demonstrated sustained plateau, and changes in fitted particle descriptors after further treatment. Together, they show why a claim of stability must identify the observed feature, processing program, and observation window. These distinctions allow preparation histories to be compared without treating every plateau as the same physical event or every subsequent structural change as a loss of dispersion stability.

### 4.2. Mixing History, Network Development, and Bulk Electrical Response

Kernin et al. [2] examine how mixing history relates to visible network development and bulk electrical conductivity. At a fixed loading of 0.05 wt% reduced graphene oxide (rGO), high-shear and medium-shear three-roll milling (TRM) produced different cured bulk conductivities. The high-shear material reached approximately 10^−3^ S m^−1^, whereas conductivity after medium-shear preparation was below the measurement range for a 70 mm-long specimen measured lengthwise. A result below this range does not imply zero conductivity. Because the comparison retains the nominal filler loading, it demonstrates that composition alone does not describe the electrical outcome at this low concentration.

The optical sequence shows how the visible rGO network developed after mixing. Following high-shear preparation, the initial rGO distribution was relatively homogeneous, without visible network connections. During cure at 120 °C, local connections developed and a clear filler path was visible after approximately 10 min. The authors describe its macroscopic shape as remaining similar through the end of cure after 12 h [2]. The later image therefore provides evidence that the visible morphology persisted after its formation. The optical observations do not continuously track individual platelets or contacts, and no independent gel measurement establishes the timing of this apparent structural stability relative to gelation.

The medium-shear comparison shows why the initial distribution matters. Its less homogeneous initial dispersion contained local agglomerates, around which network formation occurred more rapidly. Despite this faster local network development, separately cured bulk specimens prepared by medium-shear mixing had conductivity below the measurement range at 0.05 wt% rGO, whereas those prepared by high-shear mixing showed measurable conductivity. The authors attribute the conductivity difference to local connections around agglomerates after medium-shear preparation, compared with more extensive connectivity developing from the more homogeneous initial distribution after high-shear preparation. The appearance of the first visible connections therefore does not, by itself, identify which preparation will give higher bulk conductivity. The spatial distribution of those connections also matters when interpreting the comparison [2].

The advantage depended on filler loading. Above the respective percolation thresholds, medium- and high-shear specimens had very similar conductivities in the reported series. The strongest contrast concerns obtaining measurable conduction at very low loading, rather than a universal increase in conductivity with mixing intensity. Comparison with Section 4.1 shows why later observations matter: they can reveal renewed structural change, as in the clay gallery expansion reported by Kong and Park [33], or support persistence of the visible morphology, as in the rGO sequence. Because these observations concern different structural descriptors, they do not provide a common measure of particle immobilization across the two systems [2].

The optical and electrical measurements were made on separate specimens with different geometries. Optical observations used an approximately 10 µL droplet on a heated glass slide under a glass lid. Conductivity was measured by a two-point method in separately cured bulk specimens following a 120 °C/12 h program; specimen dimensions are given in Table 2. Although the optical series also includes a 12 h image, this shared observation time does not establish that the droplet morphology represents the conductive network in the bulk specimens. Scanning electron microscopy (SEM) of the final fracture surfaces provides additional structural observations, but it neither tracks contact formation during cure nor establishes a quantitative link between the optically observed morphology and the bulk conductive network [2] (Table 2).

Structural redistribution can have different consequences depending on the intended material outcome. Yourdkhani and Hubert [4] assess retention of a dispersed CNT state through an optical dispersion index, whereas Kernin et al. [2] associate rGO reagglomeration during cure with the development of conductive paths. The significance of redistribution therefore depends on whether the intended outcome is preservation of dispersion or development of conductive paths. This does not establish that reagglomeration generally improves electrical performance: the spatial distribution, loading and preparation route remain decisive parts of the comparison. The optical and bulk measurements do not isolate the individual contributions of platelet geometry, contact resistance and matrix effects to conductivity. Together, they support an association between mixing history, visible network development and bulk electrical response, but do not establish when electrical stabilization occurs relative to matrix cure.

### 4.3. Matrix Cure Markers in Assessing Structural and Electrical Stabilization

Wu et al. [59] examined how amine addition affects the development of electrical conductivity during processing. At 1.25 vol% carbon black (CB), they followed isothermal conductivity in both CB–epoxy dispersions without amine and reacting CB–epoxy–amine formulations. Both systems showed an initial conductivity increase followed by an approach to a plateau. Their responses nevertheless differed: conductivity in the dispersions without amine depended more strongly on temperature, whereas the reacting formulations approached similar conductivity levels across the tested temperatures and reached the plateau more rapidly. The authors associated this behavior with the influence of amine and/or curing on initial network development and its subsequent restriction.

The comparison with the hardener-free formulation studied by Yourdkhani and Hubert [4] helps distinguish the occurrence of a plateau from the influence of curing. An optical dispersion index reached a plateau without added hardener, and an electrical signal approached a plateau without added amine. In both studies, adding the curing component changed the measured material response. These observations show why the presence of a plateau alone cannot identify gelation and why the effects of the curing component must be assessed from the measured trajectories. The two observables remain distinct: optical microscopy describes resolved morphology, whereas conductivity responds to conductive pathways and their electrical characteristics. Moreover, adding amine changes matrix composition as well as enabling reaction, so the Wu comparison does not isolate crosslinking as the sole cause of the difference.

For the reacting CB–epoxy–amine system, Wu et al. [59] then compared conductivity development with a model-derived gel time. Their Table 3 gives a gel time of 9 min at 100 °C and an estimated time to reach the conductivity plateau of 12–26 min. At 70 and 50 °C, the corresponding values are 30 versus 35–60 min and 128 versus 130–160 min, respectively (see Table 2 of this review). The authors describe the criteria as correlated and link the electrical development to matrix crosslinking. An earlier sentence in the source, however, places plateau formation before gelation, contrary to these tabulated values. The comparison here follows the source’s Table 3 while retaining this unresolved inconsistency.

The gel times were estimated from DSC kinetics and a critical-conversion criterion of approximately 0.634, rather than measured by simultaneous rheology. The reported time ranges estimate when the conductivity plateau is reached; they are neither durations of a stable state nor confidence intervals. The ordering of the tabulated times therefore does not establish a statistically resolved separation between gelation and the onset of the conductivity plateau. Neither the conductivity measurements nor the DSC-based gel model tracks CB motion, so the difference between their estimated times cannot directly demonstrate particle rearrangement after gelation. The endpoint conductivity obtained after a separate room-temperature curing program also belongs to a different series and cannot be assigned to these isothermal specimens [59].

An existing physical filler network creates a further difficulty: it can obscure the rheological signature used to identify chemical gelation of the matrix. Romberg et al. [19] examined epoxy–fumed silica formulations at 70 °C using rapid measurements over a range of frequencies. Frequency independence of the loss tangent was observable in the neat resin and the formulation containing 2.5 wt% silica, but was masked at 5 and 10 wt% silica. The rheological response of the more highly filled materials already contained a substantial physical-network contribution before chemical gelation. A criterion applicable to the neat resin could consequently not be transferred unchanged to those formulations.

Time–cure superposition of the evolving loss-tangent spectra allowed Romberg et al. [19] to follow relative relaxation times and use their divergence to estimate chemical gelation. This approach provided a basis for comparing gelation criteria when frequency independence of the loss tangent was not observable. Conversion was assigned using relationships between storage modulus and conversion derived from rheo-Raman measurements. For the neat resin and the 5 and 10 wt% silica formulations, these relationships were based on earlier measurements by Romberg and Kotula [3]. The 2.5 wt% formulation was examined in a separate experiment following the earlier protocol. Conversion was therefore not measured simultaneously with the rapid frequency-dependent measurements. The reuse of the earlier rheo-Raman data creates a shared experimental basis between the two publications, which are discussed here in relation to gelation and in Section 5 in relation to physical support.

This rheological analysis provides an estimate of chemical gelation even when the response already includes a substantial contribution from the physical filler network. It does not directly image either chemical connectivity or the evolution of the silica architecture. The silica was coated with polydimethylsiloxane (PDMS). Citing their earlier work, Romberg et al. report no appreciable chemical interaction between these particles and the epoxy–amine matrix. The agreement in estimated gel conversion across filler contents should therefore not be generalized to reactive fillers [19].

Comparing this approach with that of Wu et al. [59] shows why the interpretation of a matrix cure marker depends on how it was obtained. A conversion-based gel model and a rheological relaxation criterion provide different bases for estimating gelation; neither directly tracks the structural evolution of the filler.

Identifying chemical gelation and assessing support from a physical filler network address different material questions. The network may contribute to supporting a deposited shape before chemical gelation, but its presence alone does not establish that support will persist during heating or fabrication. Section 5 examines that persistence through rheological response, shape retention, and actual processing outcomes.

## 5. Physical Support During Processing and Cure

Assessing support during fabrication requires following the material beyond its initial rheological state, through deformation and heating. Rheological characterization, recovery after deformation, shape retention and successful fabrication address different parts of this question. Earlier reviews address these relationships. Rau et al. [9] relate rheological measurements to extrusion and layer support, with attention to temperature and deformation history, while De Smedt et al. [10] discuss recovery, particle networks and the balance between printability and functionality. The following epoxy comparisons examine what the different tests establish about support through a specified processing and curing program.

Pekol et al. [29] compared a liquid-crystalline epoxy ink without silica with a formulation containing 8 wt% fumed silica. For the uncured molten formulations, non-oscillatory flow tests gave reported yield-stress estimates of approximately 0.096 Pa without silica and 184 Pa with 8 wt% silica. The authors obtained these estimates by averaging shear stress before viscosity entered a regime of reduced shear-rate dependence; the numerical test temperature is not explicitly specified for the flow curves. This contrast supports the use of silica to increase resistance to flow in the tested formulation. The contribution of a physical silica network is inferred from rheology rather than directly observed. The value of 184 Pa consequently characterizes this formulation under the measurement conditions; it does not define a general threshold for printing.

The study’s deformation experiments and heating observations answer complementary questions. Oscillatory measurements at 2000% and 0.01% strain modeled the contrasting deformation conditions associated with printing and the deposited material. These measurements characterize the rheological response in separate time sweeps at the two imposed strain amplitudes; they do not measure flow inside a nozzle or directly image network rebuilding. Separately, photographs of cast pellets heated at 170 °C, taken after 5 and 120 min, showed improved shape retention with silica [29]. This is a direct observation of macroscopic shape under heating, beyond the inference available from a yield-stress measurement, although the pellets have a different geometry and preparation history from an extruded filament.

Printing trials provided a further test of the formulation’s processability. Pekol et al. [29] used a nozzle temperature of 140 °C and a bed temperature of 90 °C, followed by curing at 170 °C for at least 4 h. Fabrication under this program, considered alongside the pellet comparison, supports a role for silica in maintaining shape before cure is complete. The evidence comes from related but distinct procedures: the rheometer imposes a model deformation history, the heated pellets test shape retention, and printing tests the ability to form an object. Together, these tests show how silica addition affected resistance to deformation and shape retention and demonstrate that the silica-containing formulation could be fabricated under the reported program.

The persistence of the rheological response during heating is examined more directly by Romberg and Kotula [3]. In a control containing 5 wt% silica but no hardener, dynamic yield stress decreased from approximately 217 Pa at room temperature to 70 Pa at 100 °C. This decrease shows why a room-temperature value alone is insufficient for assessing support during subsequent heating. In the reacting epoxy–amine formulations, temperature also changed the progress of cure. At 70 °C, vitrification limited the attainable conversion, whereas curing at 100 °C avoided that limitation. The thermal program must therefore balance the initial resistance to flow against the attainable cure conversion.

The measurement procedure matters when interpreting this balance. Romberg and Kotula [3] defined dynamic yield stress through the G′-G″ crossover during stress-amplitude sweeps. These sweeps disrupt the physical network, and the protocol includes a period for recovery between measurements; recovery was incomplete under some conditions. The resulting values describe a response to an imposed measurement and recovery history, rather than an undisturbed static load-bearing capacity. Simultaneous rheological measurements and Raman measurements of conversion relate the evolving mechanical response to reaction progress in the same experiment. An increase in dynamic yield stress during cure nevertheless reflects changes in the matrix as well as the contribution of the filler network; it does not directly establish how silica contacts rebuild.

For the reacting formulation containing 5 wt% silica, these measurements informed a program beginning at 70 °C and increasing to 100 °C at a conversion of approximately 0.36. Dynamic yield stress showed a negligible initial decrease at 70 °C and remained above its room-temperature reference after the ramp to 100 °C, before the approximate chemical gel point [3]. Delaying the higher-temperature stage therefore provided a way to limit the initial decrease in dynamic yield stress while allowing cure to proceed beyond the conversion limit at 70 °C.

Separate oven trials compared the final conversions obtained with the two-step program and the isothermal programs. The average final conversion with the two-step program was higher than that obtained by isothermal curing at 70 °C but lower than that obtained at 100 °C. The oven ramp was slower than the rheometer ramp, and the temperature change was scheduled using the expected time to reach the selected conversion; it was not triggered by real-time conversion measurements in the oven [3]. These trials therefore extend the assessment of curing progress to another processing setting. They do not establish preservation of a printed geometry, which the study did not test. The comparison with Pekol et al. [29] distinguishes rheological and conversion evidence used to design a thermal program from direct observations of shape retention and successful fabrication.

Albertini et al. [31] show that successful extrusion does not necessarily imply adequate support for subsequent layers, even when rheological tests suggest favorable flow and recovery behavior. Their biobased epoxy composites contained microcrystalline cellulose (MCC), microfibrillated cellulose (MFC) and, in some formulations, montmorillonite. Stepwise shear tests at 25 °C showed negligible thixotropy and a near-immediate return to the viscosity associated with each imposed shear rate. These tests do not resolve a time-dependent rebuilding process in the filler network. In the printing trials, some formulations could be extruded but progressively collapsed under subsequent layers. The ability to pass through the nozzle and the ability to support the growing object were therefore experimentally distinct outcomes within the same study.

A formulation containing 22 vol% MCC and 1 vol% MFC produced cylinders that retained their shape after an oven program of 40 °C for 16 h, followed by 2 h each at 80, 120 and 140 °C [31]. The before-and-after images support a qualitative assessment of shape retention through that program, without quantifying dimensional accuracy. The printed cylinders were separate from the cast specimens used to measure tensile properties. Separately cast specimens of the 22 vol% MCC/1 vol% MFC formulation had a tensile modulus of 5.34 ± 0.50 MPa, compared with 17.84 ± 1.28 MPa for the 29 vol% MCC/2 vol% MFC formulation. Values are means ± standard deviations from at least five tensile specimens per material. The 29 vol% MCC/2 vol% MFC formulation could not be extruded under the tested conditions. The formulation with the higher tensile modulus was therefore not the one that could be successfully printed under the tested conditions, and neither modulus was measured on the printed cylinders. Because both filler fractions change, the contrast cannot be assigned to MFC content alone.

Together, these studies distinguish three aspects of support: resistance to deformation measured rheologically, persistence of that response during a thermal program, and shape retention during fabrication. Pekol et al. [29] connect a formulation contrast to complementary rheological, heating and fabrication tests. Romberg and Kotula [3] show how thermal history alters the rheological response and how conversion measurements can guide a curing program, without testing shape retention in a printed part. Albertini et al. [31] demonstrate that extrusion can succeed while layer support fails, and that a larger modulus after cure does not guarantee processability. The comparison is qualitative: differences in matrix chemistry, filler type and loading, temperature, deformation protocol and specimen geometry prevent the reported yield stresses, including 184 and 217 Pa, from forming a ranking of materials or a common printability criterion.

The shape-retention observations show that the objects maintained their macroscopic form through the tested program. These observations do not establish whether other properties remain unchanged during further treatment. Section 6 examines this separate question through electrical measurements in other filled epoxy systems, comparing functional states across cure and subsequent heating.

## 6. Electrical Response Across Cure and Subsequent Heating

An electrical endpoint characterizes the measured electrical response after a particular processing history. Continuing the thermal program can reveal whether a formulation advantage persists, whether a change while hot reverses on cooling, or whether a later cycle ends at a different electrical state. Table 3 brings together these comparisons while retaining their distinct reference states. Section 6.1 first considers how the electrical response develops during cure and how the formulation comparison changes after further heating.

### 6.1. Electrical Development and Formulation Advantage Across Processing Stages

Tsujimura and Inoue [14] compared two epoxy pastes containing 26 mass% CNTs on a nonvolatile basis. In Paste 1, phenyl glycidyl ether (PGE) replaced 40 mass% of the epoxy component. Under a continuous-heating program, the two pastes showed closely similar conversion curves derived from DSC, but different electrical resistance trajectories. Resistance in Paste 1 decreased rapidly during the early part of the ramp, whereas resistance in Paste 0 decreased more gradually over a broader temperature range. Electrical resistance and the free-damped oscillatory response were recorded simultaneously; DSC was conducted separately at the same heating rate. The similar conversion curves therefore did not capture the different resistance trajectories. The earlier resistance decrease describes the trajectory under that program; it does not establish a time to reach a common conductivity threshold.

A separate endpoint experiment establishes how the formulation contrast depended on the processing stage. After curing at 80 °C for 1 h, the resistivities were approximately 3 × 10^2^ Ω·cm for Paste 0 and 2 Ω·cm for Paste 1, as read from Figure 4 of Tsujimura and Inoue [14]. Following an additional 1 h treatment at 220 °C, both were close to 0.3 Ω·cm (Table 3). The substantial initial contrast thus largely disappeared after post-cure. These approximate values indicate convergence but do not establish statistical equivalence. Tsujimura and Inoue’s Figure 4 includes error bars, but their statistical meaning and the replicate count are not specified in the reported methods or figure caption. The endpoint readout temperature is unspecified and cannot be inferred from the separate impedance protocol. The comparison therefore concerns the source-reported resistivities after the two treatments.

The later convergence does not negate the initial benefit; it shows that the benefit depends on the processing stage at which the pastes are compared. The ramp experiment describes how resistance changes during heating, whereas the endpoint experiment compares resistivity after specified treatments. Together, they do not establish that an earlier resistance decrease guarantees a lower resistivity after further heating, and they do not form a single measured trajectory through all the reported states [14].

The authors further investigate interfacial electrical behavior using electrical impedance spectroscopy (EIS) after selected curing times at 100 °C. They interpret shrinking capacitive arcs through an equivalent-circuit model as a decrease in interfacial resistance. This interpretation provides additional support for the authors’ contact-based explanation; individual CNT junctions were not directly observed. Impedance measurements were made after removal to a room-temperature environment, and microscopy characterized separately treated specimens [14].

These observations support considering binder and interfacial effects when interpreting electrical development, but do not establish identical conductive structures after post-cure. Similar cure shrinkage was inferred from conversion behavior rather than directly measured, and the same contacts were not followed through the endpoint comparison. The convergence alone does not distinguish the contributions of changes in contact resistance, particle arrangement and matrix state to the final resistivity. PGE also changes binder chemistry, so the formulation contrast cannot be reduced to cure rate alone [14].

### 6.2. Hot-State Response and Recovery on Cooling

Fukumoto et al. [5] distinguish the electrical response at elevated temperature from that measured after cooling. Their Ag/epoxy adhesive underwent a two-stage preparation and cure program of 100 °C for 1.8 ks followed by 180 °C for 0.9 ks, then cooling to 25 °C. During a subsequent 25→180→25 °C cycle, its reported resistivity increased from approximately 3 × 10^−7^ Ω·m before reheating to 4 × 10^−7^ Ω·m at 180 °C, then returned close to the initial value after cooling to 25 °C (Figure 11 in [5]; Table 3). This is a largely reversible response within the displayed cycle.

Recovery on cooling does not mean that the electrical response remained unchanged at elevated temperature. Measurements only before and after the cycle would miss the hot-state excursion, whereas a measurement only at 180 °C would miss recovery. The hot-state response and recovery on cooling address different requirements: maintaining electrical conduction at elevated temperature and recovering the initial electrical state afterward. Whether this resistivity increase is acceptable depends on the application.

The authors relate the behavior partly to thermal expansion and contact pressure, but the electrical measurements do not directly track changes at the conductive contacts. The resistivity calculation uses the specimen cross-section measured after cure. The figure caption and adjacent text disagree on the Ag surface treatment, identifying Ag(h) and Ag(o), respectively. The observed response therefore cannot be attributed to a specific Ag surface treatment. One displayed cycle does not establish multicycle durability.

### 6.3. Changed and Stable Electrical States During Further Heating

Successive cycle endpoints provide a different comparison in the Ag microflake/nanobelt–epoxy systems studied by Rivers et al. [15]. Formulations 601 and 603 share a matrix of diglycidyl ether of bisphenol A (DGEBA) cured with triethylenetetramine (TETA). According to the authors’ nominal labels, both contain 60 wt% silver microflakes, with 1 wt% nanobelts in 601 and 3 wt% in 603. The wt% and phr bases reported in the source are not reconciled; these labels do not define a comparison at constant total Ag content.

For 601 under the continuous Ramp program, tabulated resistivity increased from 1.09 Ω·cm after the first cycle to 12.84 Ω·cm after the second. During the first cycle, specimens were heated from 35 to 175 °C at 1 °C min^−1^ and cooled to 40 °C, followed by a second continuous heat–cool cycle. The minimum of 0.32 Ω·cm attained during the first heating ramp is a separate hot-state observation, not the first-cycle endpoint (Table 3 in [15]).

For formulation 603 after Hold (T_g_∞ − 30 °C), the reported endpoint resistivity instead decreased from 2.64 to 0.60 Ω·cm between the first and second cycles. Here, T_g_∞ denotes the final glass-transition temperature determined following the initial Ramp cure. The first program included an isothermal hold before heating to 175 °C and cooling to 40 °C; the second cycle heated continuously, without repeating that hold. These are successive states after a specified history, rather than repetitions of an identical program. Table 3 summarizes the selected programs and tabulated endpoint comparisons.

For formulation 603, Table S1 of the source gives Tg∞ = 120 °C. The temperature–time curves in Figure S4 (in [15]) of the source indicate holds of approximately 240 min at 90 °C (Tg∞ − 30 °C) and 160 min at 105 °C (Tg∞ − 15 °C), consistent with Figure 6 (in [15]) of the main article. These durations are graph estimates rather than explicitly tabulated values [15]. The source presents electrical trajectories averaged over three specimen replicates per formulation–program combination, but does not explicitly specify how each tabulated endpoint was calculated from the replicates. The readout temperature is also not explicitly defined for every tabulated endpoint; cooling to 40 °C does not establish that each value was measured at that temperature.

Comparing two initial histories within formulation 603 allows the role of processing history to be examined at the same nominal composition. After Hold (T_g_∞ − 15 °C), the endpoint increased from 0.38 to 1.45 Ω·cm between cycles, whereas after Hold (T_g_∞ − 30 °C) it decreased from 2.64 to 0.60 Ω·cm (Table 4 in [15]). Neither second cycle repeated the first-cycle hold. Among these two histories, the lower first-cycle endpoint therefore did not identify the lower second-cycle endpoint. The opposite directions of change at the same nominal formulation show why the initial program must be retained when interpreting the later endpoint. This comparison complements the contrast between 601/Ramp and 603/Hold, where both formulation and initial treatment differ.

This remains a comparison of complete processing histories, rather than an isolated test of hold temperature. The authors report substantial variation among replicates for the −15 °C condition, so the tabulated ordering is not a statistically established ranking of programs.

The authors interpret the broader electrical response in terms of matrix evolution, partial vitrification and changes at conductive contacts. Electrical, calorimetric and dilatometric measurements used separate specimens from shared batches under matched programs, while microscopy characterized the resulting material. The matched programs support comparison of electrical evolution with matrix behavior, but the measurements do not follow the same conductive contacts in a single specimen. The authors report that calorimetry detected no further curing during the second heating cycle. Non-detection does not prove the complete absence of reaction, but it limits an explanation that simply equates the changed electrical endpoint with additional cure conversion. The combined measurements support considering thermal and interfacial processes after cure, without establishing a unique cause of the endpoint changes.

Further heating need not produce a detectable electrical change. Rivers et al. [65] reported no detectable resistivity change during second heating scans of Ag microflake/epoxy specimens despite thermal expansion. Not every cure program listed in Table 3 was tested for every formulation; the reported absence of change should therefore not be read as a result established for every formulation–program combination. The second electrical trajectories are not displayed. The source specifies an upper-resistance-measurement limit, but not the smallest change resolvable during second heating. This therefore remains an author-reported qualitative example of stability, not an exact zero change or quantitative retention (Table 3).

The initial cure program still influenced the electrical state retained during subsequent heating. Different initial histories produced different first-heating resistivities, yet the authors reported no detectable change during the second heating scans. The absence of a later change therefore does not imply that the retained resistivity is independent of earlier processing. The observations also constrain a simple explanation based on bulk expansion: expansion occurred without a detected electrical change during second heating, and resistivity could decrease while expansion continued during first heating. The authors consequently considered partial vitrification and local stresses, without directly resolving the local contact mechanism.

The two studies [15,65] report different responses to further heating in Ag-filled epoxy systems: changed cycle-endpoint resistivities in one and no detectable change during second heating scans in the other. These results must be interpreted in relation to formulation and preceding history; their contrast alone does not isolate the factor responsible. Together with the reheating–cooling result discussed in Section 6.2 [5], they distinguish three outcomes: no detectable change during later heating, recovery after a hot-state excursion and a change in the reported cycle endpoint. None alone establishes general thermal durability.

Assessing electrical stability therefore requires specifying whether the material must retain its response while hot, recover after cooling or maintain it through a further processing cycle. The following discussion considers how these requirements relate to structural stabilization and physical support.

## 7. Discussion

### 7.1. Stability in Relation to the Intended Material State

The comparisons in Section 4, Section 5 and Section 6 show that stability should be assessed against the intended material state and the subsequent processing history. Local stabilization of a structural descriptor, physical support during fabrication, and electrical response during or after further treatment address different requirements. They do not represent a universal sequence or three stages demonstrated on the same specimens. Considering them together helps identify requirements that remain unresolved even when one assessment gives a satisfactory result.

Structural reorganization cannot be judged as beneficial or detrimental without specifying the intended material outcome. Preserving an initial dispersion and developing conductive connectivity impose different requirements. In Yourdkhani and Hubert [4], the optical dispersion index reached a plateau after an earlier change, indicating limited further change in that descriptor rather than preservation of the starting CNT dispersion. Kernin et al. [2], by contrast, associate rGO redistribution during cure with the development of conductive paths. The optical observations and electrical measurements on separately cured bulk specimens support a preparation-dependent association, not a directly measured structural–electrical trajectory. These comparisons show why assessment of a preparation route should distinguish the structural feature to be preserved from the connectivity to be developed; they do not establish that reagglomeration generally improves electrical performance.

A claim of structural stability needs to specify both its observational scope and its relevance to the intended outcome. The clay studies illustrate the first requirement: Kong and Park [33] observed renewed gallery expansion after an apparent plateau, whereas Hernandez et al. [74] reported eventual stabilization without displaying a sustained plateau interval. Neither record justifies extending stability beyond the feature and period actually resolved. The post-cure changes in fitted Ag particle parameters reported by Dell’Erba et al. [73] illustrate the second requirement. The authors interpreted those changes as rounding without uncontrolled aggregation. This interpretation illustrates why a change in a measured descriptor need not imply general destabilization. The relevant question is whether the change compromises a required structural feature. Answering it requires evidence linking that feature to the intended outcome; a plateau alone does not provide that link.

Matrix cure markers should be considered alongside structural or functional measurements to assess how the measured response relates to matrix state. An optical dispersion index reached a plateau without added hardener in Yourdkhani and Hubert [4], while conductivity approached a plateau without added amine in Wu et al. [59]. Adding the curing component nevertheless changed the responses. These observations distinguish the occurrence of a plateau from the influence of the curing component on the trajectory leading to it: a plateau alone cannot identify chemical gelation, and adding the curing component changes composition as well as enabling reaction. The resulting differences therefore cannot be attributed to crosslinking alone. In the Wu study, the model-derived gel times and estimated times to the conductivity plateau do not establish a statistically resolved difference in timing. A matrix marker can thus help assess the timing of structural or functional development but cannot replace direct evidence of the response whose stability matters.

### 7.2. Choosing Programs and Comparing Functional Outcomes

A fabrication program must provide sufficient resistance to deformation during processing while allowing cure to advance. These requirements can favor different temperature histories. In the epoxy–silica system of Romberg and Kotula [3], a lower initial temperature limited thermal weakening of the rheological response, but vitrification restricted the attainable conversion. Delaying the higher-temperature stage offered a way to address both constraints. Rheological measurements and separate oven measurements of final conversion support this approach within the studied system, but do not establish an optimum or demonstrate shape retention in a printed object.

A program’s ability to support fabrication must be assessed under the deformation, heating, and loading conditions experienced by the intended object. Pekol et al. [29] illustrate the complementary roles of rheology, shape-retention observations during heating, and printing trials, while Albertini et al. [31] show that successful extrusion can coexist with failure under subsequent layers. The latter study also shows why a higher modulus measured on cast specimens need not identify a formulation suitable for printing. Rheology and conversion can therefore guide program selection, while shape-retention and fabrication tests assess whether the intended outcome is achieved. Neither room-temperature yield stress nor cured modulus alone resolves that choice, and differences in formulation, measurement protocol, and geometry prevent a common ranking of the reported rheological values.

The electrical result used to select a formulation or program must likewise correspond to the processing stage relevant to its intended use. The PGE-modified CNT paste in Tsujimura and Inoue [14] had a substantially lower reported resistivity after the milder program, but this advantage largely disappeared after additional heating. Later convergence does not invalidate the earlier benefit; it changes its relevance when further heating is part of the intended history. The unspecified endpoint readout temperature prevents direct transfer of the reported values to a defined operating temperature.

Even at fixed nominal formulation, the lower first-cycle endpoint need not identify the lower endpoint after further processing. For formulation 603 in Rivers et al. [15], the reported endpoint resistivity increased from 0.38 to 1.45 Ω·cm after the initial Hold (T_g_∞ − 15 °C) history, an approximately 3.8-fold increase between the first and second cycles. After the initial Hold (T_g_∞ − 30 °C) history, it decreased from 2.64 to 0.60 Ω·cm, approximately 77%, reversing the numerical ordering of the two histories (Table 3). Program selection should therefore consider the remaining processing history rather than rely solely on the first endpoint. This comparison concerns complete processing histories, since neither second cycle repeated the initial hold. The reported replicate variation, particularly after Hold (T_g_∞ − 15 °C), means that the numerical ordering is not a statistically established ranking of programs. The comparison illustrates the relevance of assessment stage without isolating hold temperature as the cause of the difference.

Program assessment must also distinguish performance while hot from recovery after cooling. The largely reversible resistivity increase reported by Fukumoto et al. [5] shows that recovery of the initial value does not establish unchanged performance at elevated temperature. Rivers et al. [65], by contrast, report no detectable change during subsequent heating, while retaining resistivity differences established by earlier processing. Stability can therefore preserve a history-dependent value rather than imply convergence toward a common endpoint. The assessment must match the requirement: maintaining the electrical response while hot, recovering it after cooling, or retaining it through further processing. Neither a single largely reversible cycle nor the qualitative report of an unchanged second heating scan establishes general thermal durability.

Within the systems compared here, matrix conversion and bulk dimensional change are insufficient as sole predictors of electrical response. Similar conversion curves accompanied different resistance trajectories in Tsujimura and Inoue [14]. In the two Rivers studies, changed cycle endpoints occurred without further curing detected calorimetrically [15], while thermal expansion occurred without a detectable electrical change during subsequent heating [65]. The calorimetric non-detection does not exclude reaction below the detection limit, but the combined evidence limits explanations based solely on additional conversion or bulk expansion. Contact resistance, filler arrangement, and matrix state remain candidate contributions whose individual effects were not resolved. Program selection can therefore use the measured electrical differences without requiring a unique microscopic explanation.

### 7.3. How the Evidence Profiles Support the Comparisons

MCA profiles describe how evidence categories occur together across publications, helping distinguish what a material comparison can establish from what would be needed to explain how the result developed. P7 illustrates this distinction by combining processing-history comparisons, structural states, an operational endpoint, and a functional result. The underlying measurements may support choosing between preparation routes even when they do not resolve when or how structural change became limited. A missing trajectory therefore restricts the mechanistic interpretation without invalidating the measured difference between materials. The material conclusion still depends on the reported result, its comparator, and its conditions.

A shared profile can bring contrasting material outcomes into the same comparison. P9 groups studies that assess function during or after a separate further processing history [14,15,65], although the reported electrical outcomes differ. Its value is to identify a common type of assessment, not to predict the direction of the response or imply a shared mechanism. The better representation of P9 on Dimensions 3–4 makes this combination of evidence easier to identify on the map. Interpretation of this projection must account for its sensitivity to REV-009 and to merging FP with FE (Section 3.6; Supplementary, Figure S1).

Using profiles to connect structural and functional evidence requires a separate assessment of measurement conditions. P13 combines structural trajectories, local stabilization, and a functional endpoint, yet includes both known structure–function mismatches [75,76] and partial linkage between optical droplet observations and bulk measurements [2]. The droplet–bulk comparison supports an association between preparation history, local morphology, and bulk electrical response, but not a direct quantitative relation between the observed morphology and bulk transport. The condition qualifiers in Supplementary, Figure S2, record these distinctions separately from the MCA coordinates. Profiles identify the kinds of evidence present; qualifiers help assess whether the observations can support a joint interpretation. Neither a shared profile nor good map representation establishes that measurement conditions are comparable.

### 7.4. Measurements Directed at the Remaining Uncertainties

Assessing the persistence of a structural plateau requires observations that cover the processing history relevant to the intended material outcome. Extending the observation window can reveal renewed change after an apparent plateau, as shown by Kong and Park [33]. Comparing a descriptor before and after further treatment addresses whether the observed state persists through an additional processing stage, as illustrated by Dell’Erba et al. [73]. Such comparisons require a consistently defined descriptor and comparable measurement methods, with the sampled structural scale, temporal resolution and criterion for detectable change reported. Their conclusions concern the selected feature over the tested history. Using that feature to decide when processing should stop additionally requires evidence that it is relevant to the intended material function.

Where the question concerns the influence of matrix cure on structural evolution, the descriptor trajectory should be paired with an explicitly defined matrix transition determined independently of that descriptor under matched conditions. Reporting the transition criterion and uncertainty in the estimated times would help distinguish a resolved temporal ordering from an apparent one. Romberg et al. [19] illustrate why the transition criterion must suit the filled system: a pre-existing physical network can mask the conventional rheological signature of chemical gelation. Their alternative gel estimate addresses that measurement problem, but answering the structural question also requires a corresponding filler trajectory.

Connecting local structure to bulk function requires attention to both specimen history and the volume sampled. Following structural and functional quantities on the same specimen, or on companion specimens with an explicit basis for matching formulation, geometry and thermal history, would strengthen their connection. Where an optical cell necessarily differs from the functional specimen, the consequences of the different geometries and sampled volumes should be assessed even when the nominal formulation is the same. The droplet–bulk comparison in Kernin et al. [2] identifies this need directly.

Where a program has been inferred from rheology, its ability to preserve the manufactured shape remains to be tested. Measurements of the response after deformation and of conversion should be related to observations of the object’s geometry during deposition, subsequent heating and after cure, with the temperature program recorded. This would test whether the program informed by rheology in Romberg and Kotula [3] preserves the intended object, and distinguish extrusion from layer support as in Albertini et al. [31]. Measurements of the printed material’s mechanical properties would be needed if the claim extends to its mechanical performance. Observations during processing are needed to locate when deformation occurs, while the resulting assessment of support remains specific to the tested geometry and loading.

Electrical assessment should retain the response during heating alongside measurements after defined cooling, with readout temperature and the program of every cycle stated. Fukumoto et al. [5] demonstrate why before-and-after endpoints miss a reversible hot-state excursion; the differing first and second programs in Rivers et al. [15] show why successive cycle endpoints must not automatically be treated as repetitions of one exposure. Replicates and measurement uncertainty are needed to assess the reliability of a program comparison. For a report of no detectable change, as in Rivers et al. [65], a stated detection limit would define the magnitude of change that the measurement could resolve. Together, these measurements would distinguish recovery, endpoint shifts and an unchanged response within the detection limit over the tested history. Claims of long-term durability would require evidence over the corresponding exposure history.

Mechanistic measurements should address the alternative explanations left open by the comparisons, including changes in conductive contacts, filler geometry and matrix state. Coordinating electrical response with conversion, dimensional change and structural or interfacial observations under matched histories would help establish which changes coincide. Varying the thermal program within a formulation can help distinguish history effects from formulation differences. Comparisons with and without added hardener can test whether the response also occurs without that component, although adding hardener changes composition as well as enabling reaction. EIS can help assess an interpretation based on interfacial resistance, as in Tsujimura and Inoue [14], although the parameters obtained depend on the equivalent-circuit model. Imaging can examine geometrical changes within its spatial and temporal resolution. The purpose is to discriminate between specific explanations through appropriate measurements and controls, rather than to treat agreement between signals as sufficient evidence of a unique mechanism.

### 7.5. Comparative Contribution and Scope

Earlier syntheses already examine competition between structural development and gelation, vitrification and subsequent treatment, rheology in relation to fabrication, and electrical properties through cure and post-treatment [1,6,10,11]. The contribution of this review is to show how the choice of criterion and reference state changes the conclusion drawn from a material comparison. The selected examples distinguish preserving a dispersion from developing conductive connectivity, maintaining support during processing from advancing cure, and an advantage at one processing stage from performance after further treatment. These distinctions explain why a criterion may be useful for a specified material requirement without describing every aspect of stability. This interpretation rests on the material comparisons, with MCA providing a complementary description of the evidence available across publications.

The deliberately and iteratively selected corpus supports comparisons within the reviewed evidence, rather than estimates of how frequently particular profiles occur across the wider literature. The profiles also depend on extraction and coding without independent duplicate assessment. Author reassessment and AI-assisted examination of differences support traceability but do not replace a second coder. Because the LM/LE instruction changed between coding stages, its effect cannot always be separated from that of rereading. Coding priorities retain one representation per publication, and some associations partly reflect the category definitions, including the structural evidence required for local stabilization. Profile frequencies therefore describe neither all measurements reported in the publications nor their quality.

Publication counts also overstate the number of independent experimental contributions where studies share data: Romberg and Kotula [3] and Romberg et al. [19] partly share their experimental basis. Both alternative omissions of this pair were examined. Author-list screening across the corpus and targeted source checks are described in Supplementary, Section S2; they do not establish complete experimental independence or cover every laboratory-level relationship. These limitations restrict conclusions about the prevalence and independence of recurring profiles, while individual material comparisons must be assessed from the underlying results and conditions.

Combining measurements into a material interpretation remains limited by differences in specimens, geometries and processing histories. The condition qualifiers identify these restrictions separately from the evidence profiles, but a full MCA restricted to conditions matched to the functional specimens was not performed. The practical guidance derived from these comparisons supports evaluation of a specified material and processing program; it is not a validated procedure for transferring results to other formulations.

## 8. Conclusions

For the filled epoxy systems considered here, evidence of stability becomes useful for material selection when it is tied to the required state and the history over which that state must persist. Limited change in an observed descriptor under specified conditions and within a defined measurement window is distinct from retaining the initial dispersion or developing a desired functional state. Cure markers help interpret structural development but do not replace observations of filler stabilization, and a plateau alone does not identify gelation. Local structural stabilization, physical support and retention of function therefore require evidence appropriate to each question; none automatically establishes the others.

Processing history can alter both the state reached and the relevance of a comparison. An advantage measured after one program remains a valid result for that program, even if additional treatment reduces the difference or changes the relative outcomes. Electrical assessment must distinguish the response while hot, recovery after cooling and the state reached after further processing. Recovery after cooling does not establish stability at elevated temperature. Selection consequently depends on the stage of assessment and readout conditions, rather than on an endpoint detached from its preceding and intended further history.

MCA describes publication-level profiles of the available evidence within the selected corpus and helps identify comparisons that address related questions. These profiles help distinguish evidence for comparing resulting materials from evidence for reconstructing their development. The material conclusions still depend on the experiments and their conditions: a shared profile does not demonstrate a common mechanism, causality or agreement between measurement conditions. Comparisons across different systems remain qualitative.

The practical implication is to select the observable and comparator according to the intended use of the material, then assess the required response over the relevant deformation and thermal program. For fabrication, rheology and cure measurements can guide program selection, but shape retention must be demonstrated for the intended object under the relevant fabrication conditions. For electrical function, measurements must address the required readout state and relevant further treatment. Differences in specimen geometry and measurement conditions must remain explicit when these observations are combined. This provides a basis for choosing and checking a program within a specified system, while transfer to other formulations or histories requires separate evidence.

## Figures and Tables

**Figure 1:**
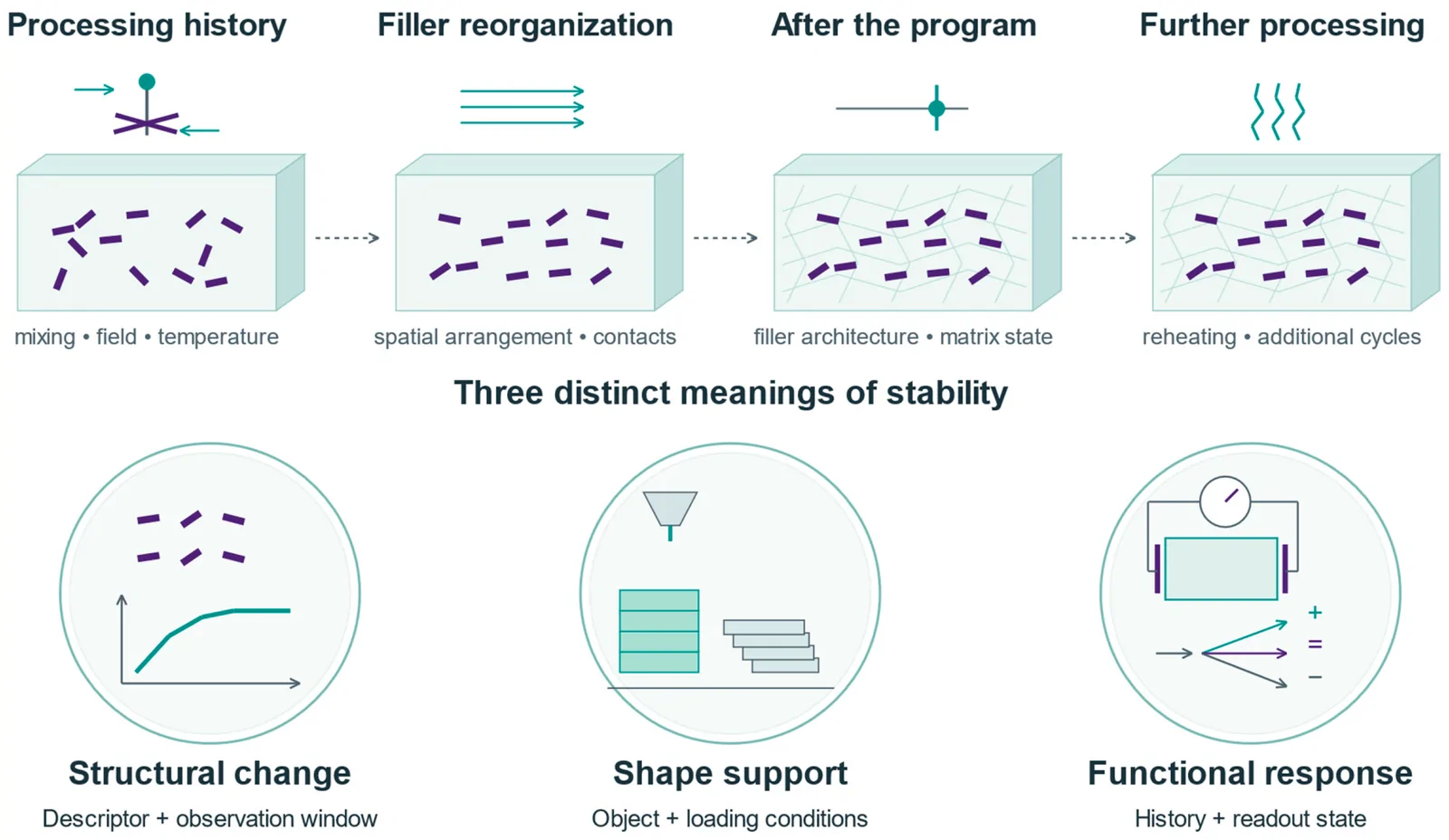
Processing history and distinct assessments of stability in particle-filled thermosets. The schematic distinguishes structural change within an observation window, shape support under specified loading, and functional response at a defined measurement state. Symbols +, = and − denote an increase, no detectable change and a decrease in the measured response, respectively. Purple segments represent filler particles, the pale network represents the polymer matrix network, and dashed arrows indicate progression through processing stages. The drawings illustrate review questions, not experimental data or a universal causal sequence.

**Figure 2:**
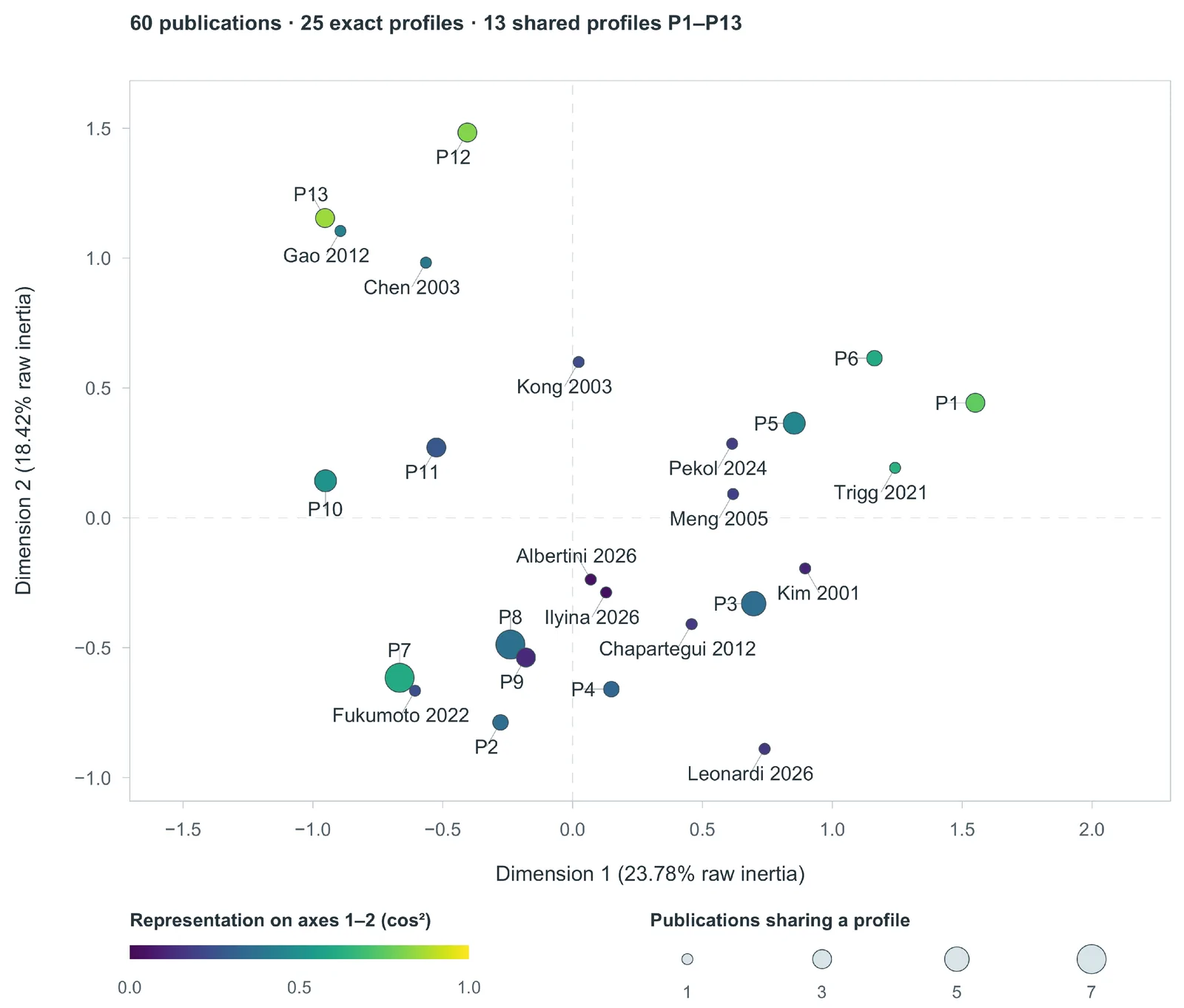
Evidence-profile coordinates on MCA Dimensions 1–2 (n = 60 publications; 25 exact profiles). Circle area encodes the number sharing an identical H/S/L/F combination, not a cluster estimate. P1–P13 identify the 13 shared profiles; other labels identify single publications. Color is summed cos^2^ in this plane, not evidence strength. Axes show raw inertia (23.78% and 18.42%; total 42.20%). Principal coordinates and category frequencies are in worksheets 08-MCA-categories and 09-MCA-publications; profile frequencies and membership are in 11-Profiles. These MCA plots were generated for this review from publication-level categorical coding of the cited studies; no graphics were reproduced or adapted from those publications [2,3,4,5,14,15,19,24,25,26,27,28,29,30,31,32,33,34,35,36,37,38,39,40,41,42,43,44,45,46,47,48,49,50,51,52,53,54,55,56,57,58,59,60,61,62,63,64,65,66,67,68,69,70,71,72,73,74,75,76].

**Figure 3:**
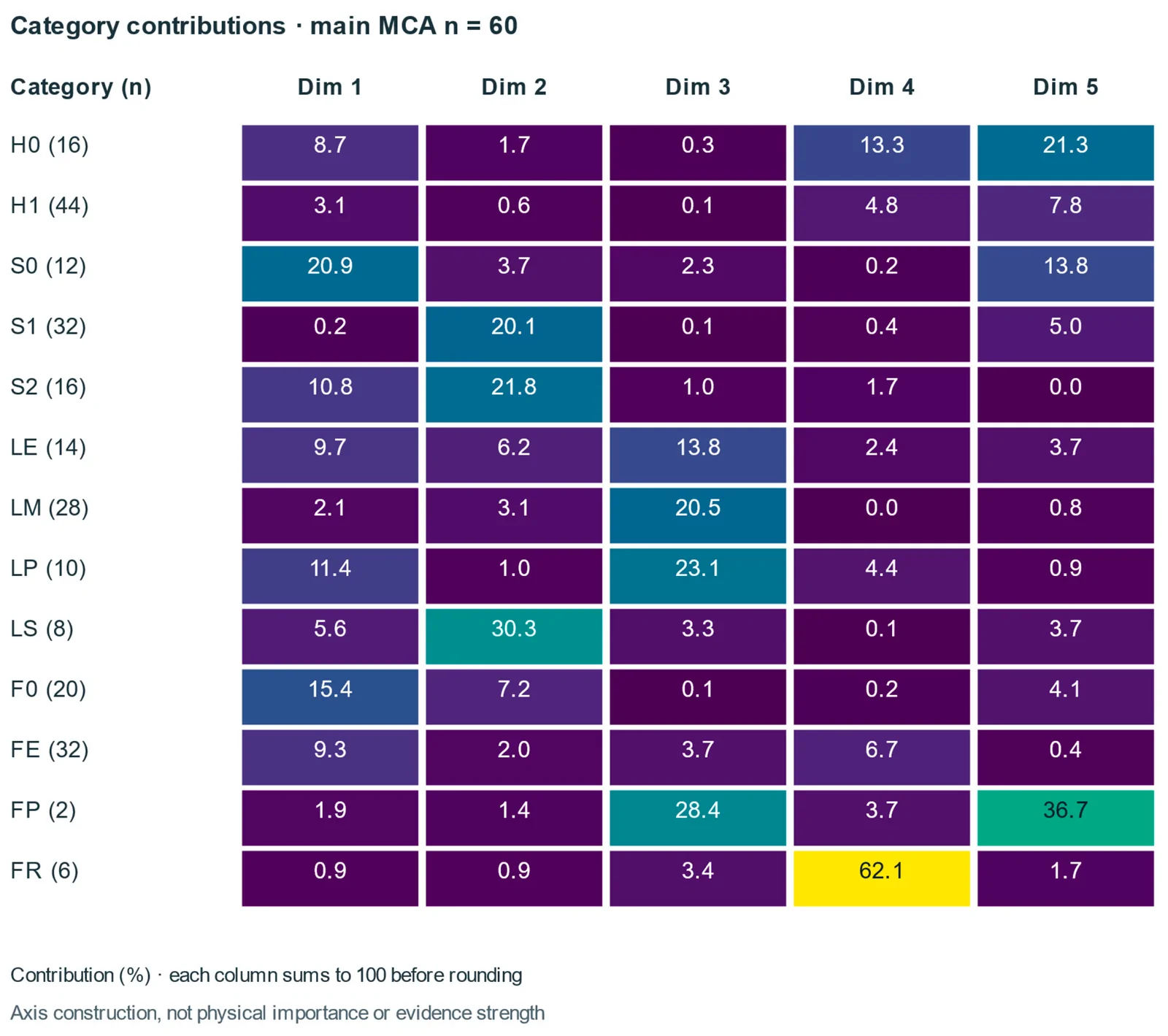
Category contributions to Dimensions 1–5 of the main MCA (n = 60). Labels include category frequencies. Values and color show contributions in percent; each column sums to 100 before rounding. Contributions indicate construction of an axis, not physical importance, evidence strength or the sign of a coordinate. The five dimensions contain 78.46% of raw inertia; coordinates and cos^2^ retain the uncorrected geometry.

**Figure 4:**
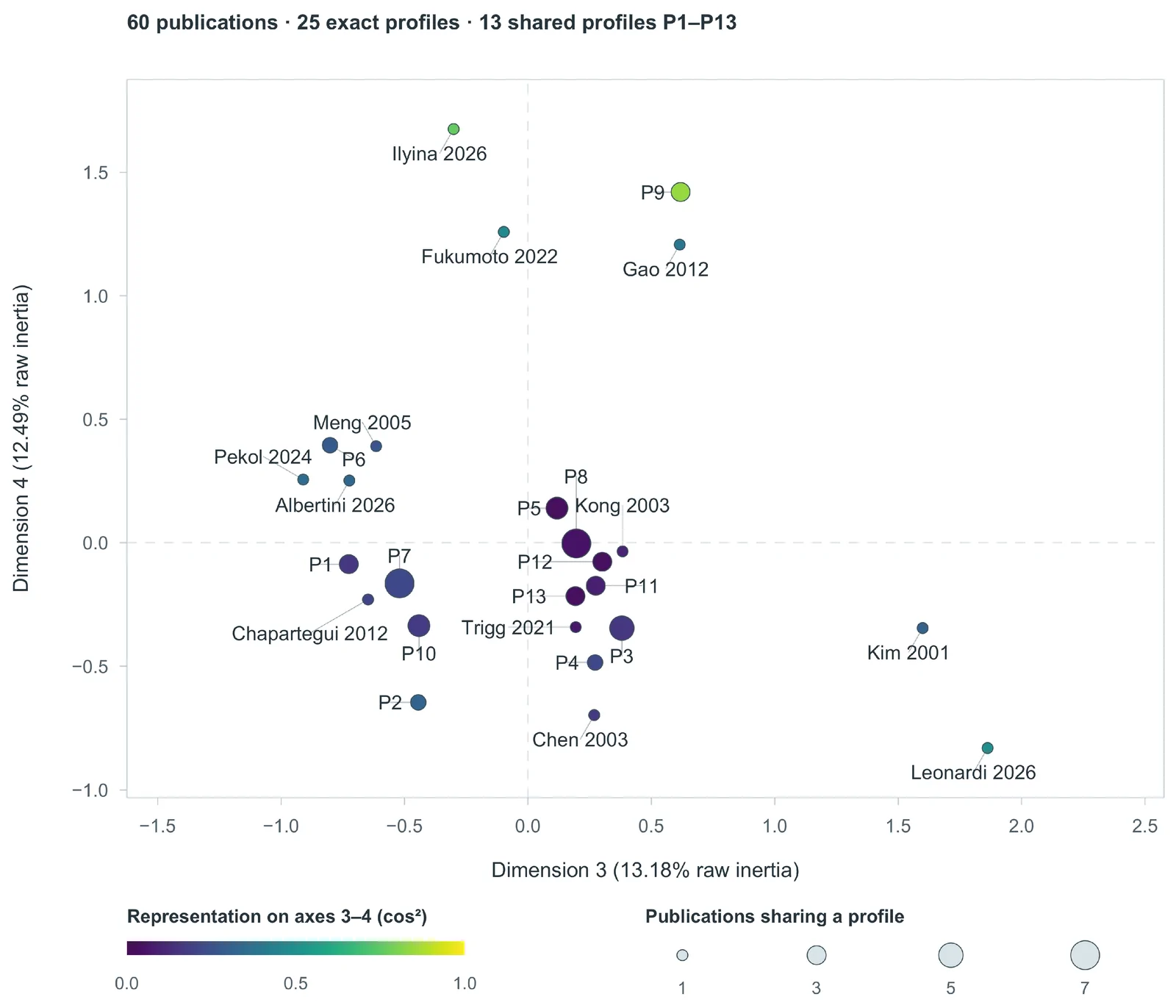
Auxiliary projection of the same 60 publications and 25 exact profiles on Dimensions 3–4. P1–P13 have the same membership as in Figure 2; circle area shows profile frequency and color shows plane-specific cos^2^. The axes contain 25.67% of raw inertia, compared with 2.22% using the separate corrected-inertia definition. The inertia adjustment does not change point coordinates or cos^2^ values. This projection is sensitive to REV-009 and to separating FP from FE; high median sensitivity similarity does not imply stability of every axis (Section 3.6). These MCA plots were generated for this review from publication-level categorical coding of the cited studies; no graphics were reproduced or adapted from those publications [2,3,4,5,14,15,19,24,25,26,27,28,29,30,31,32,33,34,35,36,37,38,39,40,41,42,43,44,45,46,47,48,49,50,51,52,53,54,55,56,57,58,59,60,61,62,63,64,65,66,67,68,69,70,71,72,73,74,75,76].

**Figure 5:**
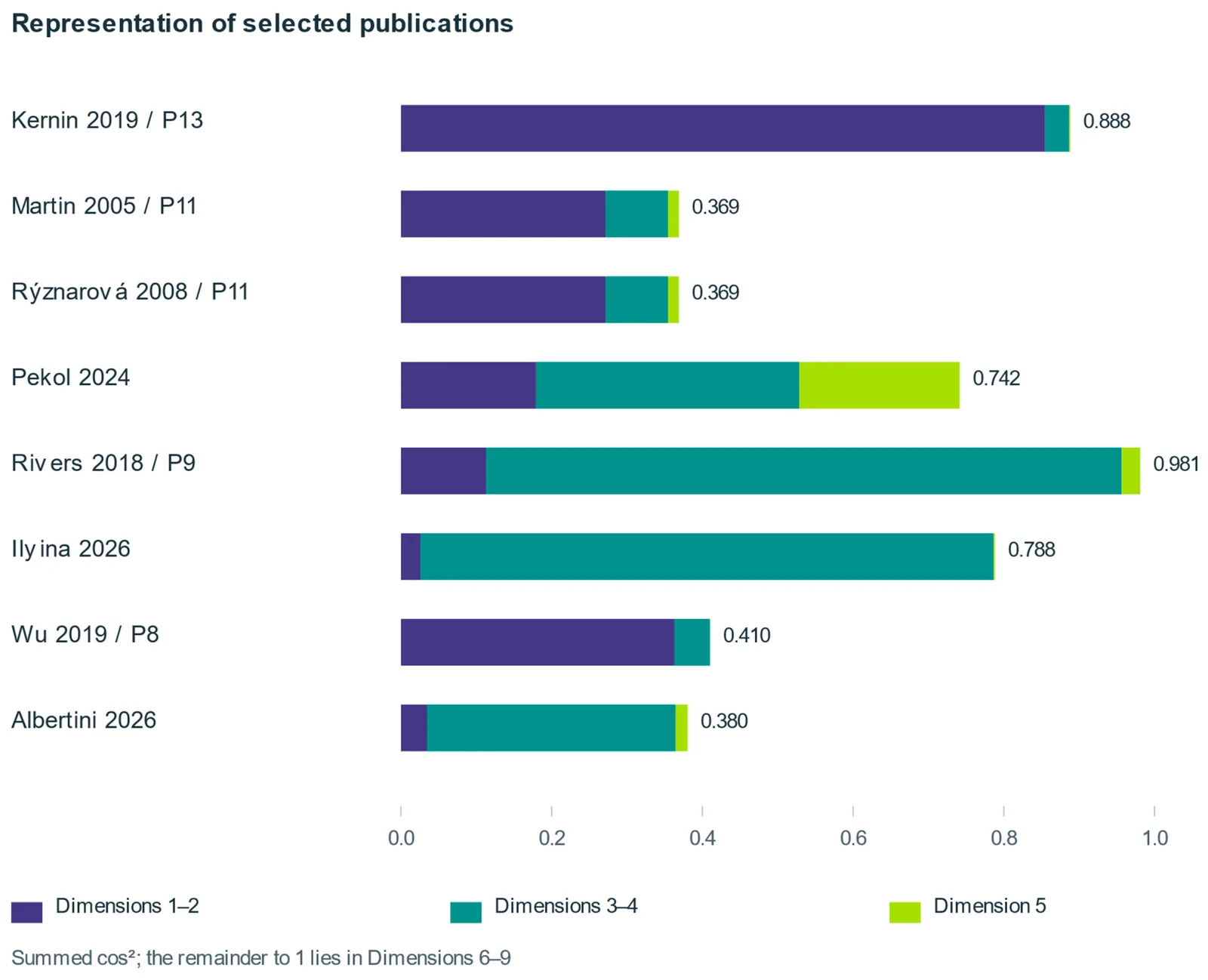
Representation of eight selected publication records and their current profiles in the main 60-publication MCA. Stacked segments show summed cos^2^ for Dimensions 1–2, Dimensions 3–4 and Dimension 5; totals cover 1–5 and the remainder to one lies in 6–9. P labels link to current shared profiles. These values measure representation in a projection, not study quality. This plot was generated for this review from the MCA results for the eight cited publications; no graphics were reproduced or adapted from those studies [2,15,29,31,32,59,70,71].

**Table 1:** Evidence-code definitions.

Field	Code	Definition	Interpretive Limit
H	H1	Deliberately compared processing histories of the same nominal formulation, including rheological experiments designed to represent processing conditions.	Composition differences alone do not establish a history comparison.
H	H0	A single history, or composition differences alone.	Different protocols of different methods do not by themselves form a history comparator.
S	S2	A displayed time or stage trajectory of a filler-architecture or population descriptor.	Reporting time-lapse measurements without presenting a structural trajectory is insufficient for S2. Differences between structural and functional measurement conditions are recorded separately and do not change the S code.
S	S1	End states, start-end, or variants without a decisive trajectory.	No decisive structural trajectory.
S	S0	Proxy without observation of the selected object’s architecture.	Images of matrix or liquid-crystalline domains do not establish the architecture of the filler network.
L	LS	A documented local limitation of change in a specified descriptor, supported by quantitative or qualitative evidence.	This code does not establish global arrest. Coding priority: LS > LP > LM > LE.
L	LP	A physical network, recovery or yield stress as the basis of support.	Measuring a rheological response does not automatically image the network.
L	LM	A measured, modeled, indirect or transferred matrix marker.	The marker’s basis and any transfer from another system are documented separately.
L	LE	An operational program or threshold, or an unverified gel postulate.	This code does not establish that gelation was independently identified.
F	F0	Characterization/proxy without a separately assessed function.	Rheological, calorimetric or optical measurements used only to characterize the material or interpret a mechanism do not by themselves establish a separately assessed function.
F	FP	Functional signal/processability during the process.	A direct functional or processability measurement, rather than characterization alone.
F	FE	Function measured after a defined manufacturing program.	Cooling is not required.
F	FR	Function also during or after a separate further thermal history or treatment.	Cyclic loading, DMA or immersion alone do not establish FR; the code does not measure quality or durability.

H: history; S: structure; L: stabilization basis; F: function. Codes describe the selected object and the measurements reported in a publication. They do not necessarily refer to measurements made on the same specimen under matched conditions or establish a causal chain.

**Table 2:** What reported plateaus and matrix markers establish in selected filled epoxy systems.

Material and Source	Compared Conditions/Preparation History	Measurement or Observation and Method	Observed Plateau or Outcome	Matrix Marker or Gel Estimate: Basis and Timing	Material Significance and Interpretation Limit
0.1 wt% CNT/epoxy Yourdkhani and Hubert [4]	With vs. without hardener; Case A: 70→120 °C at 2 °C min^−1^, then 120 °C isotherm.	Dispersion index from optical microscopy of approximately 25 µm-thick layers between cover glasses.	The index decreases during heating and plateaus during the isotherm in both systems. The authors report two or three repetitions of each experiment; one run is shown.	No independent gel marker for this plateau.	The optical dispersion index reaches a plateau even without added hardener. The plateau is limited to the observation window and image resolution; it does not establish complete CNT immobilization.
PMMA/epoxy/clay Hernandez et al. [74]	Solvent-assisted ultrasonic preparation (sample S2) vs. melt mixing (sample S4); early vs. late SAXS profiles during cure at 135 °C.	SAXS profiles; model-derived tactoid-thickness distributions.	Profiles and fitted tactoid distributions evolve over records extending to 30–36 min. The authors report eventual profile stabilization; the displayed endpoints do not establish a sustained plateau interval. [a]	Reported gel time: ≈15 min at 135 °C; the determination method is insufficiently described.	SAXS samples a limited range of structural sizes; large agglomerates may remain undetected. Complete exfoliation throughout the sample is not established. The reported post-gel interpretation is limited by the unspecified gel method.
Ag nanoparticles formed in epoxy Dell’Erba et al. [73]	Early vs. late visible-light exposure at 22–25 °C; separate comparison before/after post-cure at 150 °C for 1 h, with SAXS measured at room temperature.	Measured SAXS profiles from a 1 mm-thick reacting layer; particle parameters derived from SAXS models.	Profiles reported as nearly unchanged beyond 420 s. Post-cure slightly reduces fitted Rg and brings the Porod exponent closer to 4. [b]	Estimated gel conversion (α ≈ 0.03) from DGEBA/BF_3_·MEA cured thermally at 140 °C, applied to the photochemical conversion curves. Gelation was not independently measured in the SAXS series.	Selected particle descriptors change after post-cure, while the authors report no uncontrolled aggregation. This is not evidence of wholesale structural destabilization; particle parameters are model-derived.
0.05 wt% rGO/epoxy Kernin et al. [2]	High- vs. medium-shear TRM; optical cure at 120 °C (including a 12 h image); separate bulk cure at 120 °C for 12 h.	Optical network evolution in approximately 10 µL droplets under a glass lid; two-point conductivity measured lengthwise on separately cured 3 × 12.7 × 70 mm specimens.	High shear: visible network develops during cure; bulk conductivity ≈ 10^−3^ S m^−1^. Medium shear: conductivity below the measurement range for a 70 mm-long specimen measured lengthwise.	No independent gel measurement in the optical series.	Mixing history differentiates both network development and bulk function at this loading. Different geometries prevent direct quantitative transfer; below range does not mean zero.
1.25 vol% CB/epoxy–amine Wu et al. [59]	Isothermal cure at 100, 70 and 50 °C; electrical timing compared with the model prediction at each temperature.	Four-point DC conductivity in approximately 100 µm-thick coatings; separate isothermal DSC measurements with a model-derived critical-conversion criterion (α ≈ 0.634).	Reported time estimates for reaching the conductivity plateau: [c] 100 °C: 12–26 min 70 °C: 35–60 min 50 °C: 130–160 min	Model-derived gel time: 100 °C: 9 min 70 °C: 30 min 50 °C: 128 min	Table 3 (in [59]) of the source places plateau estimates after model-derived gel times; the authors describe them as correlated. Distinct criteria do not prove post-gel particle motion or a statistically resolved time separation. [c]

Notes. Plateau refers to the stated descriptor and observation window, not stability at every structural scale. Structural, kinetic and functional results may come from separate specimens or series, as indicated. [a] Hernandez et al. [74]: the source inconsistently assigns the 30 and 36 min endpoints to samples S2 and S4; only the combined range is retained. [b] Dell’Erba et al. [73]: no duration of stability beyond the displayed record is inferred. [c] Wu et al. [59]: values follow the source’s Table 3, although an earlier sentence places plateau formation before gelation; this inconsistency remains unresolved. The reported ranges estimate the time to the conductivity plateau; they are neither confidence intervals nor plateau durations. Abbreviations and symbols: CB, carbon black; CNT, carbon nanotubes; DC, direct current; DSC, differential scanning calorimetry; PMMA, poly(methyl methacrylate); rGO, reduced graphene oxide; SAXS, small-angle X-ray scattering; TRM, three-roll milling; α, conversion; Rg, radius of gyration; ≈, approximate value.

**Table 3:** Electrical response to additional heating: formulation contrasts, reversibility, and cycle endpoints.

Material, Formulation and Source	Previous Cure Program	Further Thermal History	States Actually Compared	Electrical Result	Significance of the Comparison and Principal Limit
CNT/epoxy, Paste 0 26 mass% CNT Tsujimura and Inoue [14]	80 °C for 1 h.	Additional heating at 220 °C for 1 h.	Four-probe resistivity of specimens printed on glass (reported dimensions: 5 × 76 mm × 30 µm), after initial cure vs. after additional heating; compared with Paste 1 at each stage. Endpoint readout temperature is unspecified.	ρ: ≈3 × 10^2^ → ≈0.3 Ω·cm	The initially much higher resistivity approaches that of Paste 1. Approximate convergence does not establish statistical equivalence or identical CNT networks.
CNT/epoxy, Paste 1 26 mass% CNT; PGE-modified binder Tsujimura and Inoue [14]	80 °C for 1 h.	Additional heating at 220 °C for 1 h.	Four-probe resistivity of specimens printed on glass (reported dimensions: 5 × 76 mm × 30 µm), after initial cure vs. after additional heating; compared with Paste 0 at each stage. Endpoint readout temperature is unspecified.	ρ: ≈2 → ≈0.3 Ω·cm	The initial advantage over Paste 0 largely disappears. PGE also changes binder chemistry; this comparison does not isolate cure rate.
Ag/epoxy adhesive Fukumoto et al. [5]	100 °C for 1.8 ks, then 180 °C for 0.9 ks; cooled to 25 °C.	One reheating–cooling cycle: 25 → 180 → 25 °C.	In situ four-point resistivity of an adhesive line on a PCB, with a 10 mm voltage-sensing length and cross-section measured after cure: 25 °C → 180 °C → 25 °C.	ρ: ≈3 × 10^−7^ → ≈4 × 10^−7^ Ω·m → near the initial value	The hot-state increase largely reverses on cooling and does not by itself indicate permanent degradation. Ag surface treatment is unresolved because the caption and text disagree. Replicate n is unspecified for this cycle; one displayed cycle does not establish multicycle durability.
Ag microflake/nanobelt–epoxy, formulation 601 Rivers et al. [15]	Ramp: 35 → 175 °C at 1 °C min^−1^; cool to 40 °C.	Second continuous heat–cool cycle; heating at 1 °C min^−1^ to 175 °C, then cooling to 40 °C.	First- vs. second-cycle endpoint (ρ∞) for 601/Ramp; in situ four-wire measurement in an 11 × 11 mm mold, nominal thickness 0.5 mm. Resistivity uses thickness measured after cure; the temperature assigned to each tabulated endpoint is unspecified.	ρ∞: 1.09 → 12.84 Ω·cm Three specimen replicates per formulation–program combination; endpoint uncertainty is not specified	The reported second-cycle endpoint is higher than the first. These endpoint values are distinct from the hot-ramp minimum and do not directly establish contact rupture.
Ag microflake/nanobelt–epoxy, formulation 603 Rivers et al. [15]	35 °C → Hold (T_g_∞ − 15 °C) or Hold (T_g_∞ − 30 °C) → 175 °C; heating at 1 °C min^−1^; cool to 40 °C. For 603, Tg∞ = 120 °C; approximate holds: 160 min at 105 °C or 240 min at 90 °C, read from source Figure S4 (in [15]) (see Section 6.3).	Second continuous heat–cool cycle, without the initial hold; heating at 1 °C min^−1^ to 175 °C, then cooling to 40 °C.	First- vs. second-cycle endpoint (ρ∞) within 603 for each initial Hold history, also comparing histories at each endpoint. In situ four-wire measurement in an 11 × 11 mm mold, nominal thickness 0.5 mm; resistivity uses post-cure thickness. Endpoint readout temperatures are unspecified.	ρ∞, first- → second-cycle endpoint: Hold (T_g_∞ − 15 °C): 0.38 → 1.45 Ω·cm Hold (T_g_∞ − 30 °C): 2.64 → 0.60 Ω·cm Three specimen replicates per formulation–program combination; endpoint uncertainty is not specified [15]	Reported endpoints shift in opposite directions and reverse their numerical ordering between histories. Substantial variation among replicates was reported, particularly for Hold (T_g_∞ − 15 °C); the ordering is not a statistically established ranking of programs. This comparison concerns complete histories and does not isolate hold temperature.
Ag microflake/epoxy: Ac0, Ac3, Ac2E1 and Ag90 Rivers et al. [65]	Ac0, Ac3 and Ac2E1: continuous ramp to 175 °C, or a 75 °C/300 min or 135 °C/60 min dwell before 175 °C. Ag90: ramp or 75 °C/300 min dwell only. Heating: 1 °C min^−1^.	Cooling, then second heating at 1 °C min^−1^ without an isothermal dwell.	In situ four-wire electrical response during second heating of previously cured specimens in an 11 × 11 mm probe-mold, following the formulation–program combinations listed here.	No detectable resistivity change reported (qualitative; no numerical Δ)	Further heating need not change the electrical response despite thermal expansion. Author-reported qualitative result; second-heating electrical trajectories are not displayed, and sensitivity to change is unspecified. The upper measurement limit does not establish the smallest detectable change.

Notes. ρ denotes resistivity; ρ∞ denotes the source-reported cycle endpoint. Arrows follow the states listed in each row; ≈ indicates approximate graph readings. Original units are retained. ks = 10^3^ s; n denotes specimen replicates per formulation–program combination. Further qualifications are discussed in Section 6.1, Section 6.2 and Section 6.3.

## Data Availability

The extracted data, publication-level coding, numerical results and analysis code supporting this review are provided in the Supplementary Materials. The original studies from which the evidence was extracted are identified in the reference list and supplementary publication register.

## Supplementary Materials

The following supporting information can be downloaded at: https://www.mdpi.com/article/10.3390/polym18212704/s1, The supporting information comprises Supplementary_Materials.docx, containing supplementary methods and Figures S1–S3 and Tables S1–S4; Supplementary_Data.xlsx, containing the search record, publication register, extracted evidence, codebook, publication-level coding, MCA results and sensitivity comparisons; and Supplementary_Code.zip, containing analysis inputs, code, numerical outputs and reproduction instructions. Figure S1 presents the sensitivity of MCA to alternative coding and corpus composition. Figure S2 presents condition linkage in the 60-publication MCA corpus. Figure S3 summarizes search and selection routes. References [77,78,79,80,81,82] are cited in the supplementary publication register.
