# Supplementary material for: The Shape of Stability: Processing History and Functional Response in Thermosets, with a Focus on Filled Epoxies
Source: Polymers (Basel). 2026 Nov 1;18(21):2704. doi: 10.3390/polym18212704 (PMC13765754; doi:10.3390/polym18212704)
Supplement: Supplementary file 1 [file polymers-18-02704-s001.zip › Supplementary_Materials.pdf]

Supplementary Materials

The Shape of Stability: Processing History and Functional Response in Thermosets, with a Focus on Filled Epoxies

This supplement provides additional methodological details, explains limitations of the source evidence, and reports MCA diagnostics. The MCA results and their interpretation are presented in Section 3 and Figures 2–5 of the main text. Figure S1 summarizes the sensitivity analyses, and Figure S2 shows condition linkage for structure–stabilization and structure–function comparisons. Figure S3 summarizes the search and selection routes.

Companion files. Supplementary_Data.xlsx contains 15 worksheets with navigation in 01-Contents: evidence and coding for 63 publications, the main 60-publication MCA, coding-check findings, first paired assignments and reassessment agreement, profile frequencies, corrected inertia, and the search-selection register. Supplementary_Code.zip supplies the final evidence base and configuration, analysis code, complete numerical outputs, figure-data generators and reproduction instructions. Worksheet names below refer to the workbook; package paths are relative to the archive root.

S1. Eligibility, Searching and Selection

S1.1. Scope and Eligibility

The review covered filled thermosetting systems, predominantly epoxies, and included bismaleimide, liquid silicone rubber, and thiol–ene systems identified through the searches. Resins without hardener, inks, and pastes were eligible as controls or as materials studied at specific processing stages. Phases added to the matrix or generated in situ included particles, platelets, fibers, and droplets. The scope extended from preparation and processing during cure to further thermal or aging treatments. Relevant observations included structural descriptors tracked over time or compared between states, physical support or processability, matrix markers, and functional measurements.

Complete reporting of formulation, loading, geometry, comparators, processing history, and readout conditions was not required for inclusion. Missing or unresolved details, such as an unspecified readout temperature, an undocumented dwell time, or an unreconciled unit basis, limit comparison and interpretation. The scope and essential selection principles are documented alongside the queries in 02-Search.

S1.2. Searches and Coverage

Scopus was searched on 8–10 September 2026 and Web of Science Core Collection on 14 September. Worksheet 02-Search lists the 14 recorded query strings, search dates, and filters; whitespace in the recorded strings has been normalized. In Scopus, Q1–Q2 targeted earlier reviews, Q3–Q5 formed the main search for primary studies, Q6–Q9 were targeted supplementary searches, and Q10 served as a saturation check. Web of Science W1–W3 targeted primary studies and W4 targeted reviews. Duplicate occurrences were removed

within the main Scopus and Web of Science screening sets. The searches and supplementary checks overlap, so their record counts are reported separately rather than summed as a single deduplicated total.

Database searches were supplemented by limited citation tracking through the OpenAlex and Semantic Scholar APIs and by targeted web searches. Citation tracking started from selected publications, with related papers considered together where applicable. Only a subset of citing publications was screened in some forward searches. Previously identified candidates were also assessed without a new search. The resulting evidence corpus was deliberately assembled and should not be treated as exhaustive.

The 63-publication analytical corpus was assembled by September 14, 2026. A targeted check of earlier reviews on 17–18 September helped clarify the review's contribution relative to previous syntheses; no empirical studies were added to the corpus. The published review by Rau et al. (2023) [1] is used in the discussion of prior reviews. Some other background sources were not fully accessible. Limited access was not taken as evidence that a source did not address a topic.

S1.3. Screening Rules and Search Limits

Scopus screening used seven relevance groups. Priority for full-text assessment and possible coding was given to records reporting direct structural evidence, indirect dynamic evidence closely related to cure, or mechanistic support with incomplete temporal coupling. The remaining groups comprised two lower-priority candidate groups, background or adjacent evidence, and out-of-scope material. These groups supported screening priorities rather than an assessment of study quality.

The Web of Science screening set contained 423 records after removal of five duplicate occurrences: 74 reviews, 13 matches to the existing library, and 336 new primary-study candidates. Of the 336 candidates, 274 were excluded at the initial screening stage, 34 were assigned to possible narrative support, 24 were prioritized for full-text assessment, and four were assigned low priority pending targeted assessment. The initial exclusion reasons concerned the material or phenomenon studied, synthesis rather than observation, final-state evidence, or insufficient temporal linkage to cure. These were stage-specific selection judgments, not uniform eligibility thresholds for the entire corpus.

All 24 priority records and one separately identified companion paper received full-text assessment. Eight priority publications entered the coded corpus. The other assessed papers were assigned supporting or companion roles, or were not retained. Search closure followed assessment of this priority set and limited citation tracking; it did not establish that every eligible publication had been identified or selected.

A targeted full-text check of the four lower-priority records found three outside the selected material scope and one topically eligible. The latter, WOS-263, remained outside the purposively selected analytical corpus. Worksheet 15-Selection distinguishes these outcomes from the initial screening statuses; none of the four records was added to the coded corpus.

The recorded Scopus queries restrict Q1–Q2 to reviews published from 2010 onward and Q3–Q5 to articles published from 2000 onward. Q6–Q7 contain no year limit. Q8–Q10 specify English-language articles published in 2000–2026, but the journal source-type filter SRCTYPE(j) appears only in Q8–Q9, not Q10. Web of Science was searched across All years. English was the intended language restriction. The primary-study exports contain journal articles and proceedings records, while W4 contains reviews. Worksheet 02-Search reports the recorded restrictions separately from the document types and languages present in each export.

Screening priorities and full-text assessment determined selection and analytical role. Detailed material comparisons were selected after extraction and coding; publications not selected for detailed discussion remained in the coded corpus. Figure S3 summarizes the routes to the corpus, and worksheet 15-Selection provides the record-level decisions and their assessment stages. The single-author workflow and AI assistance are described in Section 2.6 of the main text.

S2. Corpus and Analytical Units

The 63 coded publications are listed in 03-Publications with authors, years, titles, DOIs, analytical roles, MCA membership, and notes on related publications. REV identifiers link the evidence, coding, and numerical tables. The numbers in these identifiers do not indicate the size of the corpus.

The corpus comprises 55 empirical studies, six empirical controls and two models. Sixty empirical publications (54 studies and six controls) with complete H/S/L/F codes enter the main MCA with equal mass. REV-049 remains in the review and 61-publication empirical evidence base with unresolved L and is excluded from defining the main axes. Two 61-publication scenarios assume LE or LM for REV-049; neither assignment is treated as confirmed. All other H/S/L/F assignments are the same as in the main analysis. The two models remain outside MCA; the six controls are excluded only in the designated sensitivity variant.

The unit of analysis for material comparisons is a specific result together with its comparator, formulation, geometry, process stage, and readout conditions. The MCA unit is the publication-level evidence profile. Publications are not assumed to provide independent experimental replications. REV-033 [2] reuses the rheo-Raman storage-modulus–conversion relationships for 0%, 5%, and 10% fumed silica from REV-048 [3], while adding the measurement for 2.5% fumed silica and optimally windowed chirp experiments. These are distinct publications with partly shared experimental data, and both entries in 03-Publications document this overlap.

Both alternative omissions of the confirmed REV-033/REV-048 pair were examined; no member was selected for exclusion because it gave a more favorable result. Removing REV-033 or REV-048 yields axes-3–4 Procrustes similarity of 0.994353 or 0.994459. Whole-publication omission also removes unique information and is therefore an influence check, not perfect experiment-level deduplication.

Author lists for all 63 publications were screened for matching surnames and initials, including abbreviated initials. Thirteen potential pairs were checked in the source publications; five involved different full given names. In addition to REV-033/REV-048, shared or probable shared authorship linked REV-007/REV-015, REV-008/REV-024, REV-009/REV-038, REV-013/REV-046, REV-019/REV-028, REV-023/REV-024, and REV-032/REV-054. Shared authorship or a common procedure was not treated as evidence of data reuse.

REV-007/REV-015 links a simulation study outside MCA to a later experiment. REV-032 cites the earlier Ac0 result from REV-054 as a comparator for a new hybrid-composite series; that citation is not a new independent measurement of Ac0. REV-008 explicitly reuses conductivity data from a 2009 paper (DOI 10.1007/s10853-009-3434-7) outside the corpus. No additional pair was confirmed to reuse a measurement series within the corpus. Source locations for the Rivers comparator and the reused conductivity data are recorded in 03-Publications. The author-based screening and targeted source checks do not establish complete experimental independence or cover every laboratory-level relationship.

The related-publication notes also identify thematic links between REV-072 and REV-095 and between REV-081 and REV-092, a conference record associated with REV-086 that is not counted as a second MCA observation, and a model related to REV-089 outside the corpus.

S3. Extraction and Evidence Traceability

Data extracted from the full texts included material composition and geometry, processing conditions, structural methods and temporal resolution, stabilization markers, functional measurements, comparators, values, units, and source locations. Selected evidence is recorded in 04-Evidence, with observations, interpretations, and limitations distinguished. Worksheet 06-Coding contains the rationale for each of the four codes and the available source locators. If a rationale lacks a specific locator, consult the corresponding REV entry in 04-Evidence; this does not guarantee a precise source location for every code. Missing locators and unreported conditions were not inferred. PDF page numbers count from the first page of the consulted file, including any cover page. Printed page numbers, figure numbers, and section identifiers are provided where available.

The targeted locator check covered 25 publications: REV-001, -002, -003, -005, -009, -012, -019, -023, -025, -026, -027, -031, -032, -033, -038, -040, -042, -043, -044, -048, -051, -052, -054, -081, and -088. The selection includes assignments selected for clarification, all eight LS records, both FP records, and selected structural, printing, and electrical examples used in the main text. Thirty-seven rationale-specific locators were checked and supplied. This was a targeted check of the stated assignments and comparisons, not a new extraction or validation of every field in all 63 publications.

For REV-051 [4], the displayed SAXS profiles continue to change up to the recorded endpoints at 30–36 min. The authors report eventual stabilization, but these endpoints demonstrate neither a sustained plateau nor full exfoliation. For REV-038 [5], post-curing slightly reduces the fitted radius of gyration and shifts the Porod exponent toward 4. The authors interpret

these changes as particle rounding without uncontrolled aggregation, rather than wholesale structural breakdown. For REV-012 [6], estimated times to plateau and model-derived gel times refer to different criteria that the authors correlate. The discrepancy between the source text and table is retained in the evidence record. The reported ranges are neither plateau durations nor confidence intervals. They do not establish a statistically resolved separation between the two times or provide direct evidence of post-gel particle motion.

For the electrical comparisons, convergence between formulations after annealing (REV-019 [7]), a largely reversible electrical change while hot (REV-005 [8]), and changes in electrical endpoints after an additional cycle (REV-032 [9]) are distinct observations. The report of no detectable change during second heating in REV-054 [10] remains qualitative because the second-heating electrical trajectories are not displayed and sensitivity to change is unspecified. The stated upper measurement limit does not establish the smallest detectable change. Worksheet 04-Evidence records the unspecified readout temperatures and the unresolved description of Ag(h)/Ag(o) surface treatment. For formulation 603, the temperature–time curves in source Figure S4 indicate holds of approximately 240 min at 90 °C and 160 min at 105 °C. These are graph estimates. The source supplement also provides conversion and dimensional-change trajectories; these do not directly resolve individual filler-contact changes.

The wt% and phr bases were not interconverted where the source did not reconcile them. Approximate values read from figures were not treated as exact measurements. No pooled effect, common retention percentage, or ranking across filler systems was derived, and no new digitization was performed. These limitations also apply to the discussion in the main text.

S4. Coding and Agreement of Conditions

S4.1. Publication-Level Profiles

Definitions are provided in 05-Codebook. Worksheet 06-Coding contains the current codes, selected objects, condition qualifiers, rationales and locators for all 63 publications. REV-049 has one unresolved field, so its L cell is blank and explicitly documented rather than assigned L0. Worksheet 12-CodingChecks reports findings from the author reassessment and targeted follow-up. Worksheet 13-Reassessment retains the initial codes paired with the author reassessment and the agreement calculated before resolution of differences. The following rules clarify category interpretation.

H1 includes rheological experiments that deliberately compare processing histories of the same nominal formulation. Different protocols used for different measurement methods do not, by themselves, establish a processing-history comparison. S2 requires a displayed trajectory of a selected architecture or population descriptor over time or across processing stages; a statement that time-lapse acquisition was used is insufficient. Differences from the conditions used for functional specimens do not automatically downgrade S; these differences are recorded by the condition qualifiers.

The priority orders LS > LP > LM > LE and FR > FE > FP determine which category is retained when more than one applies; they do not rank evidence strength or durability. LS concerns

local limitation of change in a named descriptor, including qualitative observations reported by the source authors. The description must distinguish author-reported stabilization from a plateau resolved in the displayed series. LS does not establish global arrest. A transient plateau followed by further change within the same program is insufficient for LS. The basis and transfer limits of any matrix marker remain relevant even when LS or LP takes priority. LE includes an unverified claim of gelation, whereas L0 denotes no reference point and does not occur in the analyzed data.

A matrix result qualifies as LM when available evidence identifies a state or transition serving as a stabilization reference under specified process or observation conditions. Reporting Tg, DSC, viscosity or a model alone is insufficient. The connection may be the source authors' interpretation or documented coder inference. The coder may interpret a result, apply an explicit model, or transfer a marker, provided that the supporting evidence and relevant process or observation conditions are traceable. Expected curing alone is not evidence of a marker. Measured, modeled, indirect and transferred markers remain admissible. Record the source observation, interpreted matrix state, role as reference, assumptions and transfer limits. LM does not require direct evidence of filler arrest or matched conditions for every measurement. The rules for assigning categories in field F do not impose additional requirements for LM.

LE requires its own documented program, threshold or unverified gel postulate. Failure to establish LM is not automatically LE; unresolved or inaccessible information is not automatically L0. Apply the priority order only to supported categories.

Rheological, calorimetric, or optical measurements used only to characterize the material or interpret a mechanism do not by themselves establish a separately assessed function. FE does not require cooling. FR requires a separate further history; cyclic testing or immersion does not automatically meet this requirement. FE and FR publications may also contain in-process functional measurements.

Rheology alone does not image a filler network, and observations of a different object cannot substitute for structural evidence about the selected object. REV-001 [11] has a single H1/S0/LP/FE profile based on the silica-network and shape-support evidence; observations of liquid-crystalline domains are not included in that profile. H0 is retained as a sensitivity alternative.

For REV-051 [4], LS records author-reported local SAXS stabilization, subject to the source limitations described in Section S3. REV-023 [12] similarly reports that the CNF network remained stable after 30 min but does not specify how long it was subsequently observed.

S4.2. Condition Linkage

Worksheet 06-Coding records structure–stabilization (S–L) and structure–function (S–F) qualifiers separately, with notes on condition-related limitations for the 60 main-MCA publications. Figure S2 uses six categories: locally matched evidence (LC), partial linkage (PC), known mismatch (MM), linkage not established from the extracted information (NE), a specific

gap in protocol reporting confirmed in the checked source (ND), and no adequate pair (NA). NE and ND are display equivalents of the workbook labels UE and UR, respectively. For a structural series, local matching concerns the observation protocol and local stabilization of its descriptor. It does not imply that conditions match across all measurements in the publication. Condition-linkage qualifiers describe the documented relation between measurements separately from the L rationale; a different rationale does not by itself establish different measurement conditions. The qualifiers describe the documented scope of linkage and do not independently validate every marker transfer.

Partial linkage may involve related conditions or companion specimens whose comparability has not been fully established; it does not automatically indicate a flaw in the study. A known mismatch limits the transfer of conclusions between conditions without invalidating either observation. NA indicates that no adequate pair is available for comparison; it does not indicate a mismatch. S–L and S–F counts cannot be added to determine the number of complete evidence chains. A full analysis restricted to conditions matching those used for functional specimens was not performed because the extracted information was insufficient to support consistent alternative coding across the corpus.

S4.3. Author Reassessment and Resolution of Coding Differences

Sixteen of the 61 empirical publications were sampled without replacement using Python random.Random(seed).sample with seed 18432632524427526023. Reading order used a separate seed, 2213529039655371106. Neutral identifiers and separate storage hid earlier codes during the first round, but memory and AI access prevent a claim of full blinding. Full texts, available supplements, the codebook, and a blank form were supplied. Missing source material was recorded rather than replaced by AI summaries.

OA-01/REV-050 became a training and procedure-development example and was excluded from comparison without drawing a replacement. The comparison therefore covers the 15 remaining publications from the random sample and two additional targeted records, REV-009 and REV-051. REV-027 and REV-001 met the targeted criteria but had already been randomly selected and were counted only once. OA-02/REV-052 contributed its first pre-feedback codes; later training feedback was kept separately. I performed both the initial coding and the reassessment; no independent second coder participated.

The LM/LE instruction was clarified on 9 October 2026, between the initial coding and further author reassessment. The clarification described in Section S4.1 was introduced at this stage and may affect category application. Its effect cannot always be separated from that of rereading.

All four fields were assessed in each record. All 68 first reassessment assignments were completed without interpretive AI assistance.

Before adjudication, agreement between the initial coding and first author reassessment for H/S/L/F was 11/15 (73.3%), 11/15 (73.3%), 6/15 (40.0%), and 13/15 (86.7%), respectively, among the 15 remaining publications from the random sample. For the two additional targeted

records, agreement was 2/2, 2/2, 1/2, and 0/2 in the same order. Complete profiles agreed in 3/15 and 0/2 records, respectively. Unweighted Cohen's κ and nominal Krippendorff's α were calculated separately for H/S/L/F in the 15-publication group and in the combined set of 17, retaining all categories present in either assessment, including L0 (Table S4). The combined set includes the two targeted records and is not a wholly random sample. These coefficients describe agreement between stages, not independent inter-rater reliability or repeatability under unchanged instructions.

Cohen's κ uses the category proportions in each assessment stage separately. Nominal Krippendorff's α uses pooled category counts and a finite-sample correction for expected disagreement. The predominance of H1 contributes to the lower chance-corrected agreement for H despite 73.3% observed agreement in the 15-publication group. The low agreement for L reflects differences between assessment stages whose causes cannot be separated from the instruction clarification described above.

After recording and comparing the paired assignments, I examined 22 differences in 14 publications against the sources with AI assistance and made the final coding decisions. The differences concerned the selected object, comparison scope, category priorities, and interpretation, including the LM/LE boundary.

A targeted follow-up outside the comparison examined REV-015, -016, -017, -032, -035, -041, -045, -049, -050, -086, and -091; L remained unresolved for REV-049. These follow-up records were not added to the sample or agreement denominators. Worksheet 12-CodingChecks reports the findings and final rationales, while 13-Reassessment preserves the 68 initial–reassessment pairs and pre-resolution agreement. Later decisions did not replace the first reassessment assignments in the agreement calculations.

Table S1. Human and AI roles in coding checks.

Stage	Author role	AI role and limitation
Preparation	Selected the procedure	Organized sources, neutral IDs and forms; organizational masking only
Author reassessment	Recorded source-based first assignments	68 fields assessed without interpretive AI assistance
Training	Retained first OA-02 codes separately	OA-01 excluded; subsequent suggestions not counted as unassisted ratings
Adjudication	Made final coding decisions after reviewing the sources and reasoning	Assisted source and rule interpretation; not an independent second coder
Targeted follow-up	Assessed the selected records; left L unresolved for REV-049	Outside comparison; did not increase sample or agreement denominator

S5. MCA Results and Sensitivity

S5.1. Specification and Representation

MCA followed the standard indicator-matrix formulation [13]. The implementation used Python/NumPy; the R package described in that methodological reference was not used. The

complete indicator matrix G has $n = 60$ publication rows and 13 category columns for $q = 4$ active variables, H/S/L/F. Each row contains four entries equal to one, with no missing active codes. Publication masses are $1/n$ and category masses are p_j/q, where p_j is the observed proportion of publications in category j. The standardized entries are $Z_{ij} = (G_{ij} - p_j)/\sqrt{(nqp_j)}$. Singular value decomposition of Z yields nine nonzero dimensions; squared singular values are the eigenvalues. Their sum is the total raw, uncorrected inertia, $(13 - 4)/4 = 2.25$.

Filler group and geometry, property domain, year, analytical role, and condition qualifiers are supplementary descriptors and do not determine the axes. Category centroids and descriptive η^2 values were calculated for each categorical descriptor on each of the first five axes. For a given axis, η^2 is the between-category sum of squares divided by the total sum of squares of publication coordinates. Pearson and Spearman correlations describe associations between year and publication coordinates. These are descriptive summaries, not significance tests. The outputs are stored in the package results/ directory as supplementary_centroids.csv, supplementary_eta2.csv and year_correlations.csv; supplementary_frequencies.csv reports category counts for the retained descriptors.

All nine eigenvalues, raw inertia percentages and variable contributions are in 07-MCA-axes. Worksheet 08-MCA-categories reports category frequencies, principal coordinates, $\cos^2$ and contributions for axes 1–5; 09-MCA-publications reports publication coordinates and $\cos^2$. Full nine-axis outputs are results/main60_axes.csv, results/main60_categories.csv, results/main60_scores.csv and results/main60_variable_contrib.csv. Worksheet 11-Profiles and results/profile_frequency.csv report the 25 exact profiles and their membership; results/profile_membership.csv maps each REV identifier to its profile. Table S2 provides an accessible frequency summary.

Raw inertia quantifies dispersion of the coded profiles, not variation in a material property. A category's contribution to an axis is its mass multiplied by its squared principal coordinate on that axis, divided by the axis eigenvalue and expressed as a percentage. Category contributions sum to 100% per axis. A variable's contribution is the sum of the contributions of its categories. A point's $\cos^2$ on an axis is its squared coordinate on that axis divided by the sum of its squared coordinates across all dimensions. Summing $\cos^2$ across the axes of a plane measures how well the point is represented in that projection. The $\cos^2 < 0.20$ flag used for the first plane is descriptive, not a significance threshold or an assessment of study quality.

Adjusted inertia is reported separately from raw geometry. Let λk be the squared singular values of the standardized indicator matrix, $q = 4$ active variables and $J = 13$ categories. Benzécri's adjustment is $bk = [q/(q-1) \times (\lambda k-1/q)]^2$ for $\lambda k > 1/q$ and zero otherwise [14]. Normalizing by Σbk gives $100bk/\Sigma bk$. Greenacre's denominator is $IG = q/(q-1)[\Sigma\lambda k^2 - (J-q)/q^2]$, with shares $100bk/IG$ [14] (Section 4, Eq. 6). These shares need not sum to 100%; the residual is not assigned mechanically to axes 5–9. Worksheet 14-Inertia reports results for all nine axes in the main analysis and both scenarios under both normalization schemes. Main60 has $\Sigma b = 0.198031$ and $IG = 0.251116$; its Greenacre shares are 76.64% for 1–2 and 2.22% for 3–4. Corresponding shares are 75.64%/2.35% under scenario61_LE and 76.18%/2.24% under

scenario61_LM. Only the first four eigenvalues exceed 1/4. This threshold is a heuristic, not a significance test. Coordinates, contributions and $\cos^2$ are not rescaled by this reporting adjustment.

Identical H/S/L/F combinations coincide by construction; no clustering algorithm determines the number of profiles. The main 60-publication solution has 25 profiles, including 13 shared and 12 singleton profiles. Shared labels P1–P13 follow lexicographic order of current combinations. The first two dimensions contain 42.20% of raw inertia, the first four 67.87% and the first five 78.46%. 10 publications have $\cos^2$ below 0.20 in the first plane. FR has plane-specific $\cos^2$ of 0.037 and 0.820 for 1–2 and 3–4. The second projection is retained as an auxiliary map, with its weaker corrected share and sensitivity explained in the main text.

Table S2. Frequency of exact H/S/L/F profiles in the main MCA. P labels denote shared profiles; singleton labels identify publications. Full membership and REV-to-profile mapping are in worksheet 11-Profiles.

Profile	H/S/L/F	n	REV identifiers
Trigg 2021	H0/S0/LM/F0	1	REV-018
P1	H0/S0/LP/F0	3	REV-020; REV-033; REV-046
P2	H0/S1/LE/FE	2	REV-008; REV-030
P3	H0/S1/LM/F0	5	REV-015; REV-034; REV-089; REV-091; REV-092
P4	H0/S1/LM/FE	2	REV-016; REV-047
Leonardi 2026	H0/S1/LM/FP	1	REV-009
Chapartegui 2012	H0/S1/LP/FE	1	REV-014
Chen 2003	H0/S2/LS/FE	1	REV-044
P5	H1/S0/LM/F0	4	REV-011; REV-013; REV-039; REV-053
Kim 2001	H1/S0/LM/FP	1	REV-027
P6	H1/S0/LP/F0	2	REV-031; REV-048
Pekol 2024	H1/S0/LP/FE	1	REV-001
P7	H1/S1/LE/FE	7	REV-004; REV-017; REV-021; REV-029; REV-037; REV-078; REV-081
Fukumoto 2022	H1/S1/LE/FR	1	REV-005
P8	H1/S1/LM/FE	7	REV-006; REV-012; REV-028; REV-035; REV-041; REV-050; REV-086
P9	H1/S1/LM/FR	3	REV-019; REV-032; REV-054
Meng 2005	H1/S1/LP/F0	1	REV-022
Albertini 2026	H1/S1/LP/FE	1	REV-036
Ilyina 2026	H1/S1/LP/FR	1	REV-040
P10	H1/S2/LE/FE	4	REV-003; REV-025; REV-072; REV-095
Kong 2003	H1/S2/LM/F0	1	REV-045

Profile	H/S/L/F	n	REV identifiers
P11	H1/S2/LM/FE	3	REV-024; REV-026; REV-088
P12	H1/S2/LS/F0	3	REV-002; REV-038; REV-051
P13	H1/S2/LS/FE	3	REV-023; REV-042; REV-052
Gao 2012	H1/S2/LS/FR	1	REV-043

S5.2. Sensitivity Controls

Three specified perturbations (H0 for REV-001, exclusion of six controls, and merger of FP with FE) and 60 single-publication omissions yield 63 perturbation solutions. Each is compared with the main solution in axes 1–2, 3–4 and 1–5, giving 189 comparisons. The main60, scenario61_LE and scenario61_LM solutions form three pairs compared in the same spaces, giving nine further comparisons. The resulting 198 comparisons each have three descriptive similarity measures. Each comparison uses common publication identifiers and separately centers both coordinate matrices on that common set. No alternative axes are selected to maximize resemblance. Worksheet 10-Sensitivity and results/comparisons_198.csv report the comparisons; Table S3 summarizes only the 60 leave-one-out runs, each fitted with 59 publications. The named perturbations and REV-049 scenarios are reported separately.

Spearman correlation of pairwise Euclidean distances measures agreement in the relative separations between publications. Distances are rounded to 12 decimal places before assigning average ranks to ties.

Orthogonal alignment follows the Procrustes formulation [15]. The normalized similarity used here is the sum of singular values of A^TB divided by $\|A\|_F\|B\|_F$, where A and B are the centered coordinate matrices and $\|\cdot\|_F$ denotes the Frobenius norm. It measures agreement after orthogonal alignment and normalization of overall scale. For the principal-angle comparison [16], QR decomposition provides orthonormal bases Q_A and Q_B; the smallest singular value of $Q_A^TQ_B$ is the minimum principal-angle cosine. It assesses the least similar direction between subspaces and is distinct from a point's $\cos^2$.

The analyses are exploratory. High medians do not imply stability of every direction. For axes 3–4, median Procrustes similarity is 0.998800, but omitting REV-009 gives 0.628515 and minimum subspace cosine 0.216943. FP/FE merger gives plane similarity 0.902598 while correlations of the corresponding individual axes, allowing sign reversal, are only 0.379 and 0.376; FR becomes the leading contributor to axis 3 rather than 4. In 5D, the merger gives minimum cosine 0.558, and omitting REV-027 gives 0.216. These cosines are not point $\cos^2$. Figure S1 reports selected summaries; the full table also includes comparisons showing lower similarity.

Leave-one-out quartiles use Hyndman–Fan type 7 (NumPy quantile, method="linear"): $h=(n-1)p$ with zero-based indices and linear interpolation between adjacent ordered values; IQR=Q3−Q1. The 60 perturbations are not independent experimental replications and IQR is

not a confidence interval. Other perturbations and REV-049 scenarios are not pooled with them.

The timing of specification of the initial sensitivity analyses is not established. REV-049 scenarios were specified before their calculation, but after the initial analysis and the coding uncertainty were known. The axes-3–4 checks were specified after the 1–2 and 1–5 analyses.

These controls are exploratory, not preregistered or confirmatory. No p-values or BH adjustment are supplied: the similarity metrics are descriptive. Free permutation of fields would ignore code-definition constraints; bootstrap would require a justified publication-resampling model and would not validate coding or shared-data independence.

Table S3. Leave-one-out summaries, 60 runs per space, n = 59 in each fitted variant. Minima identify the omitted publication.

Axes / measure	Median	Q1	Q3	IQR	Minimum	Omitted REV
1-2 / ρ	0.998408	0.997980	0.999129	0.001149	0.984923	REV-043
1-2 / P	0.999696	0.999396	0.999879	0.000484	0.990802	REV-043
1-2 / c_min	0.999508	0.998806	0.999787	0.000981	0.979647	REV-043
3-4 / ρ	0.996653	0.991830	0.997515	0.005684	0.768106	REV-009
3-4 / P	0.998800	0.995295	0.999538	0.004244	0.628515	REV-009
3-4 / c_min	0.997909	0.991527	0.999271	0.007744	0.216943	REV-009
1-5 / ρ	0.998947	0.998104	0.999528	0.001423	0.976324	REV-027
1-5 / P	0.999668	0.999222	0.999909	0.000687	0.916070	REV-027
1-5 / c_min	0.998872	0.995787	0.999734	0.003947	0.216260	REV-027

ϱ: Spearman correlation of pairwise distances; P: normalized Procrustes similarity; c_{min}: minimum principal-angle cosine.

Exploratory sensitivity · main MCA n = 60

	Distance rho			Procrustes P			Min. subspace cos.		
	1-2	3-4	1-5	1-2	3-4	1-5	1-2	3-4	1-5
REV-001 → H0	0.994	0.976	0.993	0.998	0.987	0.995	0.996	0.979	0.973
Without 6 controls	0.963	0.895	0.984	0.982	0.958	0.954	0.968	0.932	0.580
FP + FE → FX	0.980	0.836	0.836	0.993	0.903	0.937	0.986	0.806	0.558
Omit REV-033	0.998	0.990	0.998	0.999	0.994	1.000	0.999	0.991	0.999
Omit REV-048	0.998	0.984	0.998	0.999	0.994	1.000	0.999	0.989	0.998
LOO median (60 runs)	0.998	0.997	0.999	1.000	0.999	1.000	1.000	0.998	0.999
LOO minimum*	0.985	0.768	0.976	0.991	0.629	0.916	0.980	0.217	0.216
61: REV-049 = LE*	0.997	0.995	0.998	1.000	0.997	0.999	1.000	0.994	0.995
61: REV-049 = LM*	1.000	0.996	0.999	1.000	0.998	1.000	1.000	0.996	0.998

* Minimum may identify different omissions for each metric and space.

LOO Q1, Q3 and IQR: Table S3 and worksheet 10-Sensitivity.

REV-049 codes are scenario assumptions, not confirmed assignments.

Pair omissions are included in LOO; shown separately for the shared-data check.

Figure S1. Exploratory sensitivity of the main 60-publication MCA in axes 1–2, 3–4 and 1–5. Comparisons use common publications and fixed axis indices; Procrustes similarity allows orthogonal alignment and overall normalization. Other perturbations, both omissions of the partly shared-data pair, leave-one-out summaries and REV-049 scenarios are displayed separately. Leave-one-out comprises 60 runs with 59 publications each; medians and minima are descriptive, with full quartiles in Table S3. Minima need not identify the same omission. LE/LM for REV-049 are scenario assumptions only. High medians do not imply stability of every direction; axes 3–4 are sensitive to REV-009 and the FP/FE distinction.

Publication	Record	S–L	S–F
Pekol 2024	REV-001	NA	NA
Yourdkhani 2015	REV-002	LC	NA
Han 2021	REV-003	PC	NE
Zhu 2009	REV-004	PC	MM
Fukumoto 2022	REV-005	PC	PC
Dean 2005	REV-006	PC	PC
Sumfleth 2011	REV-008	PC	MM
Leonardi 2026	REV-009	MM	PC
Harsch 2008	REV-011	NA	NA
Wu 2019	REV-012	PC	PC
Kortaberria 2006	REV-013	NA	NA
Chapartegui 2012	REV-014	PC	MM
Furuta 2026	REV-015	PC	NA
Jie 2024	REV-016	MM	PC
Nagarajan 2021	REV-017	PC	MM
Trigg 2021	REV-018	NA	NA
Tsujimura 2022	REV-019	PC	PC
Chiou 2001	REV-020	NA	NA
Hu 2008	REV-021	PC	PC
Meng 2005	REV-022	PC	NA
Prasse 2003	REV-023	LC	MM
Martin 2005	REV-024	PC	PC
Yakovenko 2017	REV-025	PC	MM
Rýznarová 2008	REV-026	MM	MM
Kim 2001	REV-027	NA	NA
Sakaniwa 2016	REV-028	PC	PC
Li 2026	REV-029	PC	PC
Wang 2014	REV-030	PC	PC
Weißer 2023	REV-031	NA	NA
Rivers 2018	REV-032	PC	PC
Romberg 2025	REV-033	NA	NA
Sanctuary 2009	REV-034	MM	NA
Zhao 2008	REV-035	PC	ND
Albertini 2026	REV-036	PC	PC
Liu 2026	REV-037	PC	PC
Dell'Erba 2017	REV-038	LC	NA
Surendran 2021	REV-039	NA	NA
Ilyina 2026	REV-040	PC	PC
Morais 2019	REV-041	PC	PC
Belijar 2016	REV-042	LC	MM
Gao 2012	REV-043	LC	ND
Chen 2003	REV-044	LC	MM
Kong 2003	REV-045	PC	NA
Nohales 2013	REV-046	NA	NA
Liu 2019	REV-047	MM	MM
Romberg 2023	REV-048	NA	NA
Nuhiji 2013	REV-050	PC	PC
Hernandez 2007	REV-051	LC	NA
Kernin 2019	REV-052	LC	PC
Altmann 2003	REV-053	NA	NA
Rivers 2017	REV-054	PC	PC
Chen 2026	REV-072	PC	NE
Kim 2020	REV-078	PC	MM
Wu 2017	REV-081	PC	PC
Zhou 2008	REV-086	PC	PC
Wilson 2005	REV-088	MM	MM
Lagasse 2002	REV-089	PC	NA
Fukushima 1978	REV-091	PC	NA
Tiwari 2023	REV-092	PC	NA
Mao 2024	REV-095	PC	PC

S–L: structure–stabilization basis S–F: structure–function

Code	Meaning
LC	Locally matched
PC	Partially linked
MM	Known mismatch
NE	Linkage not established
ND	Specific protocol detail unreported
NA	No adequate pair

Figure S2. Condition linkage in the 60-publication MCA corpus. S–L describes how structural evidence relates to the basis for assessing stabilization; S–F describes how it relates to functional assessment. Codes denote locally matched evidence (LC), partial linkage (PC), known mismatch (MM), linkage not established from the extracted information (NE), a specific gap in protocol reporting confirmed in the checked source (ND), or no adequate pair (NA). Colors identify categories, not quality ranks. The qualifiers do not establish complete process–structure–function chains. Author/year labels identify publications; REV identifiers link to the publication list in 03-Publications. REV-049 remains in the evidence base but is omitted from this main-MCA display.

S5.3. Computational Reproduction

The package runs from its root with python3 reproduce.py after installing the dependencies listed in requirements.txt. The script run_analysis.py reads inputs/evidence_base_63.json and inputs/analysis_config.json and fits MCA by singular value decomposition of the indicator matrix for the main analysis, both REV-049 scenarios, three named perturbations and 60 leave-one-out variants: 66 fits in total. The configuration specifies membership, ordering and scenario assumptions. The evidence base retains unresolved L for REV-049; LE or LM is applied only within its respective scenario. For every fit, results/derived_inputs/ contains an indicator matrix (*_indicator.csv) identifying the actual H/S/L/F codes and publication order, and a mass table (*_masses.csv). The script also calculates sensitivity comparisons, adjusted inertia, leave-one-out summaries and the retained supplementary summaries. It does not use saved coordinates to fit the analyses.

The package requires Python 3.10 or later and NumPy 1.22 or later; results/summary.json records the environment used for each run. The wrapper reproduce.py then calls prepare_figures.py to prepare CSV data and R renderers in figures/Figure_2 through Figure_5 and figures/Figure_S1 and Figure_S2. This step does not export images. Rendering is a separate operation using base R/grid, Arial and macOS Quartz; the renderers produce editable SVG, vector PDF and 600 dpi PNG files. Figures 2–5 use the fitted coordinates, contributions and profile representation; Figure S1 uses the sensitivity results, and Figure S2 uses the condition qualifiers. In figures/Figure_S2/figure_S2_conditions.csv, S_L_input and S_F_input are input qualifier labels corresponding to display codes S_L and S_F, not earlier coding assignments. The workbook stores static results. The README gives the commands and file locations.

The code checks the input checksum, indicator-matrix algebra, equal masses, an independent Gram-matrix eigenvalue calculation, barycentric reconstruction, identical-profile coordinates, the Greenacre denominator computed directly from off-diagonal Burt blocks, the Procrustes residual identity and independent type-7 quartile interpolation. It also checks variant membership and comparison counts. Fit checks are recorded in results/fit_checks.csv, and results/summary.json summarizes the analyses and environment. These checks concern numerical implementation; reproducibility does not validate source interpretation, coding decisions, sampling completeness or independence of experimental data.

S6. Search and Selection Flow

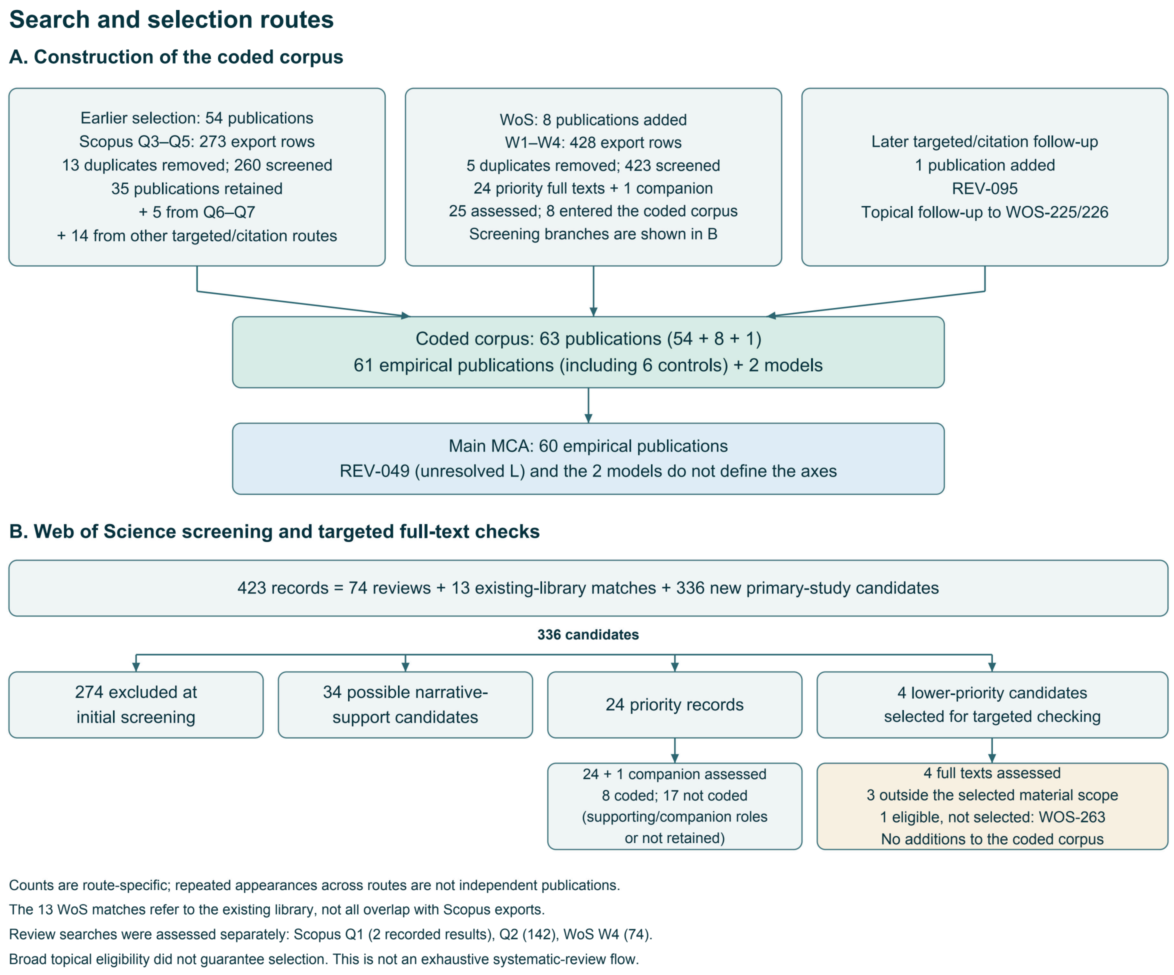

Figure S3. Search and selection routes to the coded corpus. (A) Contributions to the 63-publication corpus and the main 60-publication MCA. (B) Web of Science screening and the targeted full-text assessment of four lower-priority candidates. Counts refer to the indicated search route or assessment stage; repeated appearances across routes are not added as independent publications. The 13 Web of Science matches are matches to the existing library, not a complete count of overlap with all Scopus exports. Broad topical eligibility did not guarantee selection into the purposively assembled corpus. The diagram is a route-specific selection flow, not an exhaustive systematic-review accounting.

S7. Agreement Between Coding Stages

Table S4. Agreement between initial coding and first author reassessment, before adjudication.

Publication subset	Field	Identical	Agreement	Cohen’s κ	Nominal α
Random remainder (15)	H	11/15	73.3%	0.189	0.194
Random remainder (15)	S	11/15	73.3%	0.589	0.597
Random remainder (15)	L	6/15	40.0%	0.192	0.136
Random remainder (15)	F	13/15	86.7%	0.776	0.781
Combined set (17)	H	13/17	76.5%	0.358	0.365
Combined set (17)	S	13/17	76.5%	0.630	0.637
Combined set (17)	L	7/17	41.2%	0.206	0.156
Combined set (17)	F	13/17	76.5%	0.616	0.626

The random remainder excludes the training case REV-050. The combined set adds the targeted publications REV-009 and REV-051. All coefficients use first reassessment assignments; later decisions do not replace these values. Calculation inputs and outputs are supplied in Supplementary_Code.zip under agreement/, with corresponding results in worksheet 13-Reassessment.

References for Supplementary Materials

References are numbered independently within this supplement. The source studies for the full coded corpus are identified by DOI in 03-Publications.

1. Rau, D.A.; Williams, C.B.; Bortner, M.J. Rheology and Printability: A Survey of Critical Relationships for Direct Ink Write Materials Design. *Progress in Materials Science* **2023**, *140*, 101188, doi:10.1016/j.pmatsci.2023.101188.
2. Romberg, S.K.; Lehrman, S.; Seppala, J.E.; Kotula, A.P. Identifying the Chemical Gel Point of Epoxy-Fumed Silica Composites Using Optimally Windowed Chirp Measurements. *Rheologica Acta* **2025**, *64*, 673–685, doi:10.1007/s00397-025-01526-7.
3. Romberg, S.K.; Kotula, A.P. Simultaneous Rheology and Cure Kinetics Dictate Thermal Post-Curing of Thermoset Composite Resins for Material Extrusion. *Additive Manufacturing* **2023**, *71*, 103589, doi:10.1016/j.addma.2023.103589.
4. Hernandez, M.; Sixou, B.; Duchet, J.; Sautereau, H. The Effect of Dispersion State on PMMA-Epoxy-Clay Ternary Blends: In Situ Study and Final Morphologies. *Polymer* **2007**, *48*, 4075–4086, doi:10.1016/j.polymer.2007.05.013.
5. dell'Erba, I.E.; Martínez, F.D.; Hoppe, C.E.; Eliçabe, G.E.; Ceolín, M.; Zucchi, I.A.; Schroeder, W.F. Mechanism of Particle Formation in Silver/Epoxy Nanocomposites Obtained through a Visible-Light-Assisted in Situ Synthesis. *Langmuir* **2017**, *33*, 10248–10258, doi:10.1021/acs.langmuir.7b01936.
6. Wu, T.H.; Foyet, A.; Kodentsov, A.; van der Ven, L.G.J.; van Benthem, R.A.T.M.; de With, G. Curing and Percolation for Carbon Black-Epoxy-Amine Nanocomposites. *Composites Science and Technology* **2019**, *181*, 107672, doi:10.1016/j.compscitech.2019.05.029.
7. Tsujimura, S.; Inoue, M. Effect of Binder Chemistry on Dynamic Percolation in Electrically Conductive Carbon-Nanotube-Filled Pastes during Curing. *MATERIALS TRANSACTIONS* **2022**, *63*, 1281–1286, doi:10.2320/matertrans.mt-m2022052.
8. Fukumoto, S.; Makimoto, K.; Ohta, K.; Nakamura, T.; Matsushima, M.; Fujimoto, K. Change in Electrical Conductivity of Electrically Conductive Adhesives during Curing Process. *Journal of Materials Science* **2022**, *57*, 11189–11201, doi:10.1007/s10853-022-07305-6.
9. Rivers, G.; Lee-Sullivan, P.; Zhao, B. How Interface Compatibility Affects Conductivity Evolution of Silver Nanobelts-Filled Electrically Conductive Composites during Cure and Post-Treatments. *Physical Chemistry Chemical Physics* **2018**, *20*, 17999–18008, doi:10.1039/c8cp01875h.
10. Rivers, G.; Lee-Sullivan, P.; Zhao, B. Vitrification during Cure Produces Anomalies and Path-Dependence in Electrical Resistance of Conductive Composites. *Composites Science and Technology* **2017**, *149*, 90–99, doi:10.1016/j.compscitech.2017.06.011.
11. Pekol, C.; Furst, J.; Li, Y.; Keum, J.; Harper, D.P. 3D Printing of Thermally Responsive Shape Memory Liquid Crystalline Epoxy Networks. *ACS Omega* **2024**, *9*, 40801–40809, doi:10.1021/acsomega.4c05664.
12. Prasse, T.; Cavaillé, J.-Y.; Bauhofer, W. Electric Anisotropy of Carbon Nanofibre/Epoxy Resin Composites Due to Electric Field Induced Alignment. *Composites Science and Technology* **2003**, *63*, 1835–1841, doi:10.1016/s0266-3538(03)00019-8.
13. Nenadic, O.; Greenacre, M. Correspondence Analysis in R, with Two- and Three-Dimensional Graphics: The ca Package. *Journal of Statistical Software* **2007**, *20*, 1–13, doi:10.18637/jss.v020.i03.
14. Nenadic, O.; Greenacre, M. *Computation of Multiple Correspondence Analysis, with Code in R*; Social Science Research Network: Rochester, NY, 2005;
15. Schönemann, P.H. A Generalized Solution of the Orthogonal Procrustes Problem. *Psychometrika* **1966**, *31*, 1–10, doi:10.1007/BF02289451.

16. Björck, Å.; Golub, G.H. Numerical Methods for Computing Angles between Linear Subspaces. *Mathematics of Computation* **1973**, *27*, 579–594, doi:10.1090/S0025-5718-1973-0348991-3.
