# Supplementary material for: The Shape of Stability: Processing History and Functional Response in Thermosets, with a Focus on Filled Epoxies
Source: Polymers (Basel). 2026 Nov 1;18(21):2704. doi: 10.3390/polym18212704 (PMC13765754; doi:10.3390/polym18212704)
Supplement: Supplementary file 1 [file polymers-18-02704-s001.zip › Supplementary_Code.zip!/Supplementary_Code/figures/Figure_S1/Figure_S1.pdf]

## Exploratory sensitivity · main MCA n = 60

| | Distance rho | | | Procrustes P | | | Min. subspace cos. | | |
|---|---|---|---|---|---|---|---|---|---|
| | 1-2 | 3-4 | 1-5 | 1-2 | 3-4 | 1-5 | 1-2 | 3-4 | 1-5 |
| REV-001 → H0 | 0.994 | 0.976 | 0.993 | 0.998 | 0.987 | 0.995 | 0.996 | 0.979 | 0.973 |
| Without 6 controls | 0.963 | 0.895 | 0.984 | 0.982 | 0.958 | 0.954 | 0.968 | 0.932 | 0.580 |
| FP + FE → FX | 0.980 | 0.836 | 0.836 | 0.993 | 0.903 | 0.937 | 0.986 | 0.806 | 0.558 |
| Omit REV-033 | 0.998 | 0.990 | 0.998 | 0.999 | 0.994 | 1.000 | 0.999 | 0.991 | 0.999 |
| Omit REV-048 | 0.998 | 0.984 | 0.998 | 0.999 | 0.994 | 1.000 | 0.999 | 0.989 | 0.998 |
| LOO median (60 runs) | 0.998 | 0.997 | 0.999 | 1.000 | 0.999 | 1.000 | 1.000 | 0.998 | 0.999 |
| LOO minimum* | 0.985 | 0.768 | 0.976 | 0.991 | 0.629 | 0.916 | 0.980 | 0.217 | 0.216 |
| 61: REV-049 = LE* | 0.997 | 0.995 | 0.998 | 1.000 | 0.997 | 0.999 | 1.000 | 0.994 | 0.995 |
| 61: REV-049 = LM* | 1.000 | 0.996 | 0.999 | 1.000 | 0.998 | 1.000 | 1.000 | 0.996 | 0.998 |

* Minimum may identify different omissions for each metric and space.

LOO Q1, Q3 and IQR: Table S3 and worksheet 10-Sensitivity.

REV-049 codes are scenario assumptions, not confirmed assignments.

Pair omissions are included in LOO; shown separately for the shared-data check.
