# Supplementary material for: The Shape of Stability: Processing History and Functional Response in Thermosets, with a Focus on Filled Epoxies
Source: Polymers (Basel). 2026 Nov 1;18(21):2704. doi: 10.3390/polym18212704 (PMC13765754; doi:10.3390/polym18212704)
Supplement: Supplementary file 1 [file polymers-18-02704-s001.zip › Supplementary_Code.zip!/Supplementary_Code/figures/Figure_2/Figure_2.pdf]

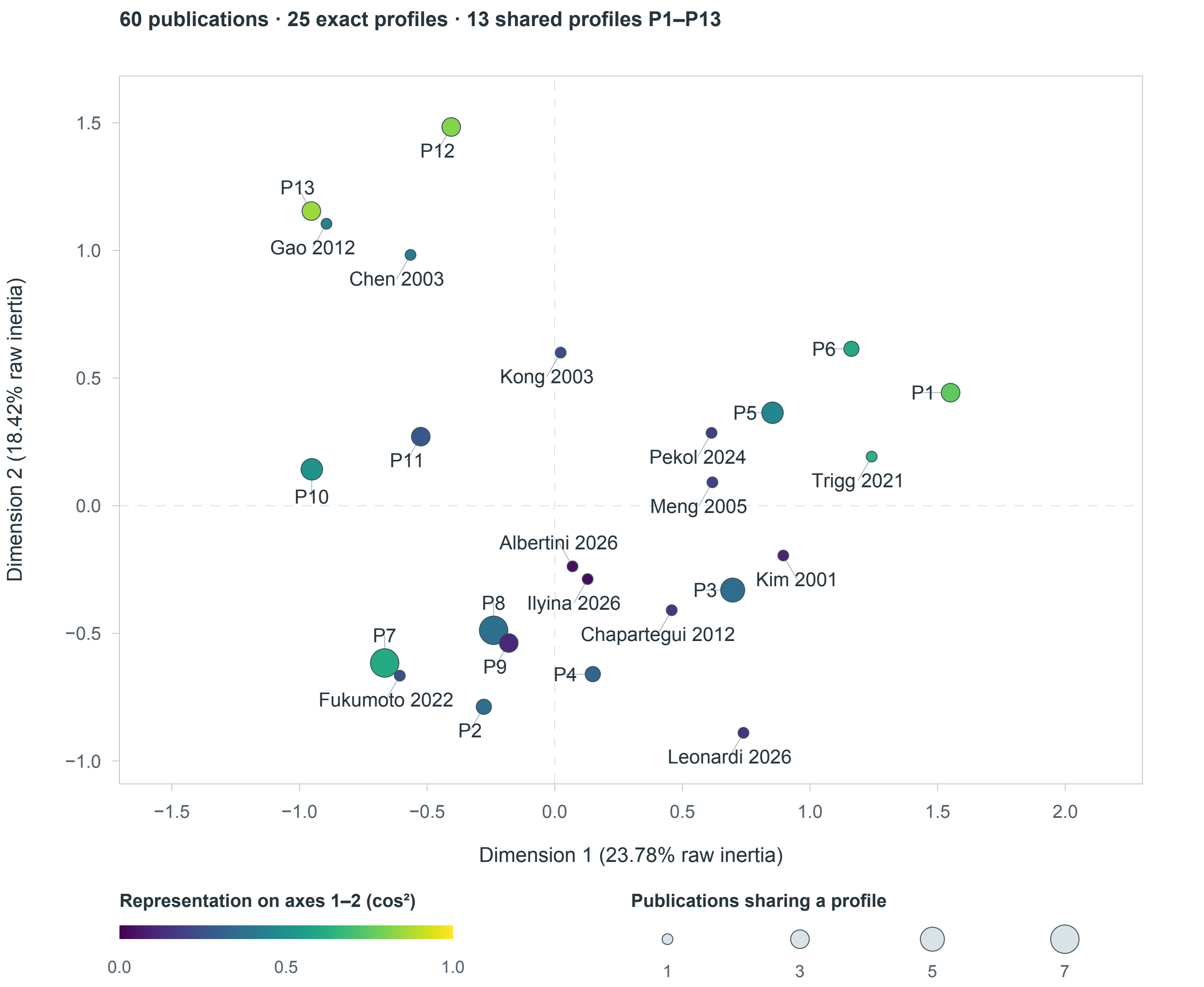
60 publications · 25 exact profiles · 13 shared profiles P1–P13
P12
P13
Gao 2012
Chen 2003
Kong 2003
P6
P1
P5
Pekol 2024
P11
P10
Trigg 2021
Meng 2005
Albertini 2026
Kim 2001
P3
Ilyina 2026
P8
Chapartegui 2012
P7
P9
P4
Fukumoto 2022
P2
Leonardi 2026
1.5
1.0
0.5
0.0
−0.5
−1.0
−1.5
2.0
Dimension 1 (23.78% raw inertia)
Dimension 2 (18.42% raw inertia)
Representation on axes 1–2 (cos²)
Publications sharing a profile
1
3
5
7
