# Supplementary material for: The Shape of Stability: Processing History and Functional Response in Thermosets, with a Focus on Filled Epoxies
Source: Polymers (Basel). 2026 Nov 1;18(21):2704. doi: 10.3390/polym18212704 (PMC13765754; doi:10.3390/polym18212704)
Supplement: Supplementary file 1 [file polymers-18-02704-s001.zip › Supplementary_Code.zip!/Supplementary_Code/figures/Figure_3/Figure_3.pdf]

Category contributions · main MCA n = 60

Category (n)	Dim 1	Dim 2	Dim 3	Dim 4	Dim 5
H0 (16)	8.7	1.7	0.3	13.3	21.3
H1 (44)	3.1	0.6	0.1	4.8	7.8
S0 (12)	20.9	3.7	2.3	0.2	13.8
S1 (32)	0.2	20.1	0.1	0.4	5.0
S2 (16)	10.8	21.8	1.0	1.7	0.0
LE (14)	9.7	6.2	13.8	2.4	3.7
LM (28)	2.1	3.1	20.5	0.0	0.8
LP (10)	11.4	1.0	23.1	4.4	0.9
LS (8)	5.6	30.3	3.3	0.1	3.7
F0 (20)	15.4	7.2	0.1	0.2	4.1
FE (32)	9.3	2.0	3.7	6.7	0.4
FP (2)	1.9	1.4	28.4	3.7	36.7
FR (6)	0.9	0.9	3.4	62.1	1.7

Contribution (%) · each column sums to 100 before rounding

Axis construction, not physical importance or evidence strength
