# Supplementary material for: The Shape of Stability: Processing History and Functional Response in Thermosets, with a Focus on Filled Epoxies
Source: Polymers (Basel). 2026 Nov 1;18(21):2704. doi: 10.3390/polym18212704 (PMC13765754; doi:10.3390/polym18212704)
Supplement: Supplementary file 1 [file polymers-18-02704-s001.zip › Supplementary_Code.zip!/Supplementary_Code/figures/Figure_4/Figure_4.pdf]

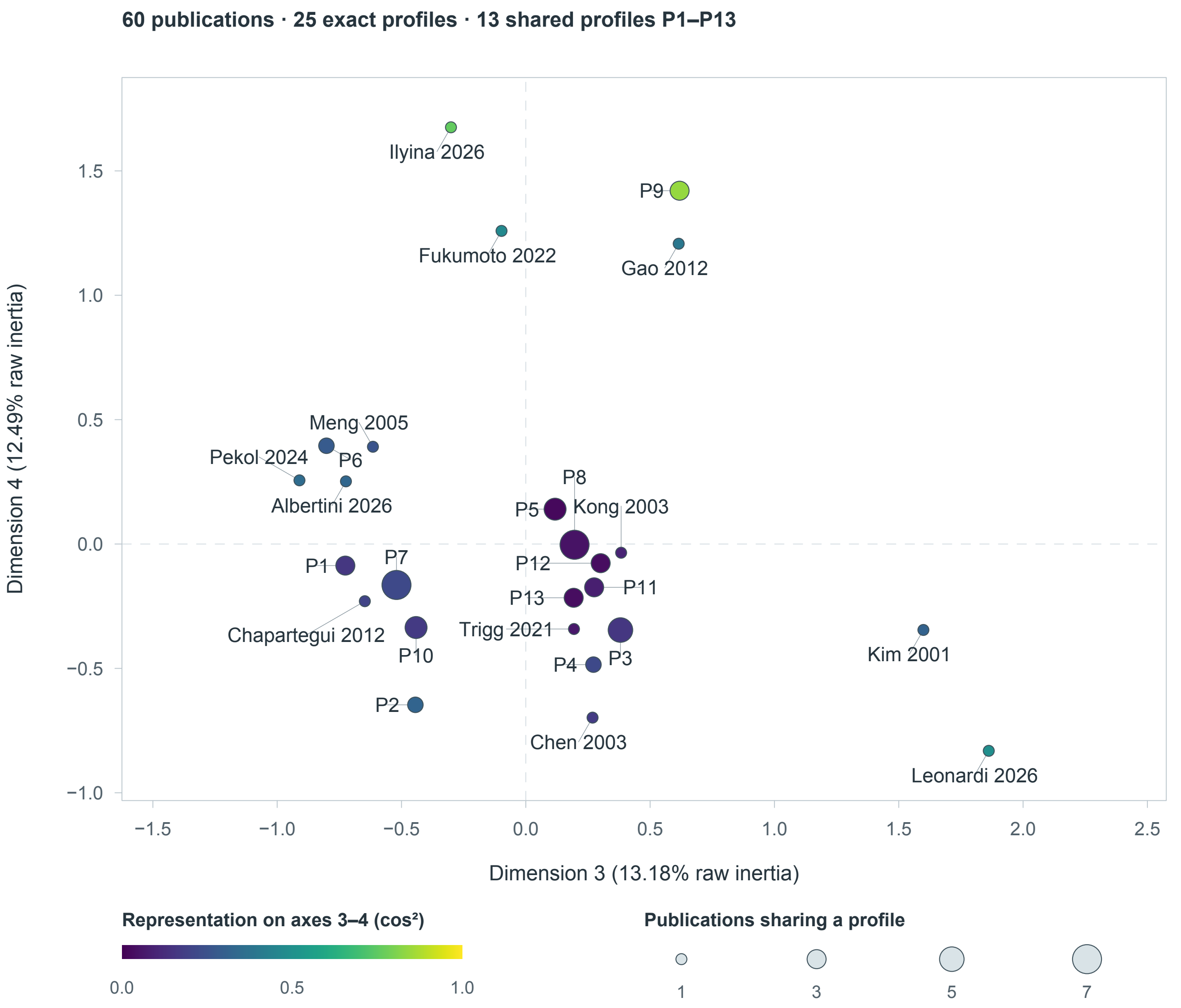

60 publications · 25 exact profiles · 13 shared profiles P1–P13
Ilyina 2026
P9
Fukumoto 2022
Gao 2012
Meng 2005
Pekol 2024
P6
Albertini 2026
P8
P5
Kong 2003
P7
P1
P12
P11
P13
Chapartegui 2012
Trigg 2021
P10
P4
P3
Kim 2001
P2
Chen 2003
Leonardi 2026
Dimension 4 (12.49% raw inertia)
Dimension 3 (13.18% raw inertia)
1.5
1.0
0.5
0.0
−0.5
−1.0
−1.5
2.0
2.5
Representation on axes 3–4 (cos²)
0.0
0.5
1.0
Publications sharing a profile
1
3
5
7
