# Supplementary material for: The Shape of Stability: Processing History and Functional Response in Thermosets, with a Focus on Filled Epoxies
Source: Polymers (Basel). 2026 Nov 1;18(21):2704. doi: 10.3390/polym18212704 (PMC13765754; doi:10.3390/polym18212704)
Supplement: Supplementary file 1 [file polymers-18-02704-s001.zip › Supplementary_Code.zip!/Supplementary_Code/figures/Figure_S2/Figure_S2.pdf]

Publication	Record	S–L	S–F
Pekol 2024	REV-001	NA	NA
Yourdkhani 2015	REV-002	LC	NA
Han 2021	REV-003	PC	NE
Zhu 2009	REV-004	PC	MM
Fukumoto 2022	REV-005	PC	PC
Dean 2005	REV-006	PC	PC
Sumfleth 2011	REV-008	PC	MM
Leonardi 2026	REV-009	MM	PC
Harsch 2008	REV-011	NA	NA
Wu 2019	REV-012	PC	PC
Kortaberria 2006	REV-013	NA	NA
Chapartegui 2012	REV-014	PC	MM
Furuta 2026	REV-015	PC	NA
Jie 2024	REV-016	MM	PC
Nagarajan 2021	REV-017	PC	MM
Trigg 2021	REV-018	NA	NA
Tsujimura 2022	REV-019	PC	PC
Chiou 2001	REV-020	NA	NA
Hu 2008	REV-021	PC	PC
Meng 2005	REV-022	PC	NA
Prasse 2003	REV-023	LC	MM
Martin 2005	REV-024	PC	PC
Yakovenko 2017	REV-025	PC	MM
Rýznarová 2008	REV-026	MM	MM
Kim 2001	REV-027	NA	NA
Sakaniwa 2016	REV-028	PC	PC
Li 2026	REV-029	PC	PC
Wang 2014	REV-030	PC	PC
Weißer 2023	REV-031	NA	NA
Rivers 2018	REV-032	PC	PC
Romberg 2025	REV-033	NA	NA
Sanctuary 2009	REV-034	MM	NA
Zhao 2008	REV-035	PC	ND
Albertini 2026	REV-036	PC	PC
Liu 2026	REV-037	PC	PC
Dell'Erba 2017	REV-038	LC	NA
Surendran 2021	REV-039	NA	NA
Ilyina 2026	REV-040	PC	PC
Morais 2019	REV-041	PC	PC
Belijar 2016	REV-042	LC	MM
Gao 2012	REV-043	LC	ND
Chen 2003	REV-044	LC	MM
Kong 2003	REV-045	PC	NA
Nohales 2013	REV-046	NA	NA
Liu 2019	REV-047	MM	MM
Romberg 2023	REV-048	NA	NA
Nuhiji 2013	REV-050	PC	PC
Hernandez 2007	REV-051	LC	NA
Kernin 2019	REV-052	LC	PC
Altmann 2003	REV-053	NA	NA
Rivers 2017	REV-054	PC	PC
Chen 2026	REV-072	PC	NE
Kim 2020	REV-078	PC	MM
Wu 2017	REV-081	PC	PC
Zhou 2008	REV-086	PC	PC
Wilson 2005	REV-088	MM	MM
Lagasse 2002	REV-089	PC	NA
Fukushima 1978	REV-091	PC	NA
Tiwari 2023	REV-092	PC	NA
Mao 2024	REV-095	PC	PC

S–L: structure–stabilization basis   S–F: structure–function

- LC Locally matched
- PC Partially linked
- MM Known mismatch
- NE Linkage not established
- ND Specific protocol detail unreported
- NA No adequate pair
