# Supplementary material for: The Shape of Stability: Processing History and Functional Response in Thermosets, with a Focus on Filled Epoxies
Source: Polymers (Basel). 2026 Nov 1;18(21):2704. doi: 10.3390/polym18212704 (PMC13765754; doi:10.3390/polym18212704)
Supplement: Supplementary file 1 [file polymers-18-02704-s001.zip › Supplementary_Code.zip!/Supplementary_Code/figures/Figure_S3/Figure_S3.pdf]

Search and selection routes

A. Construction of the coded corpus

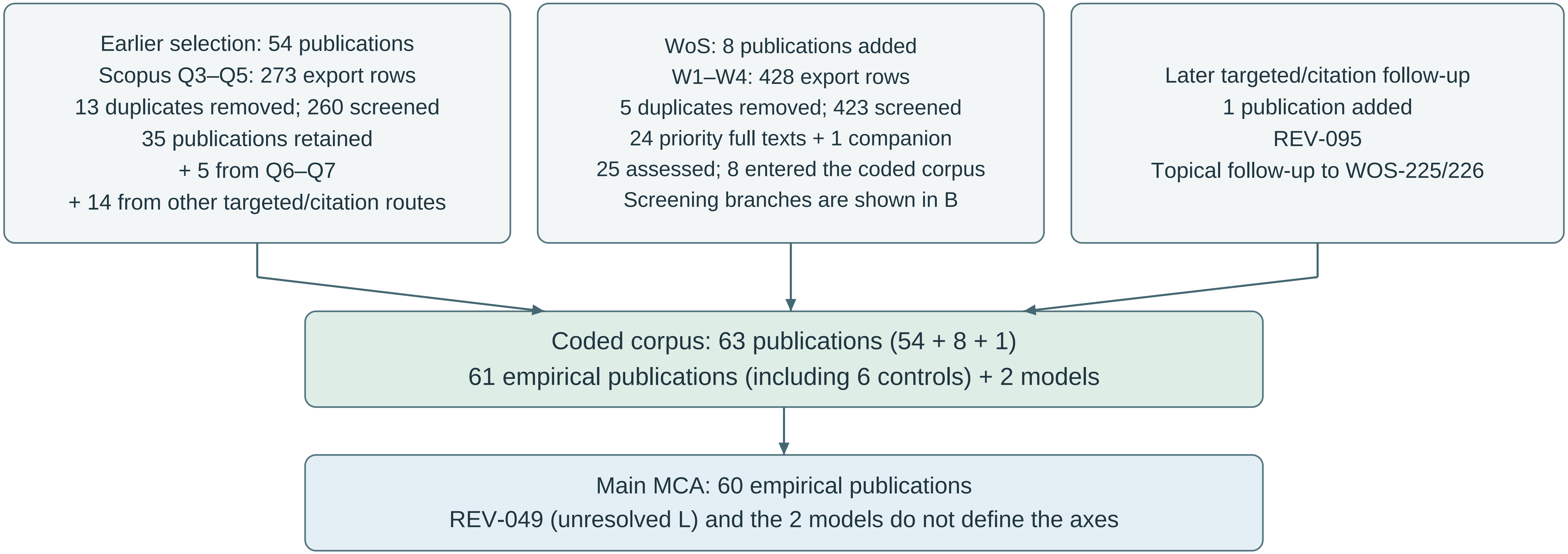

B. Web of Science screening and targeted full-text checks

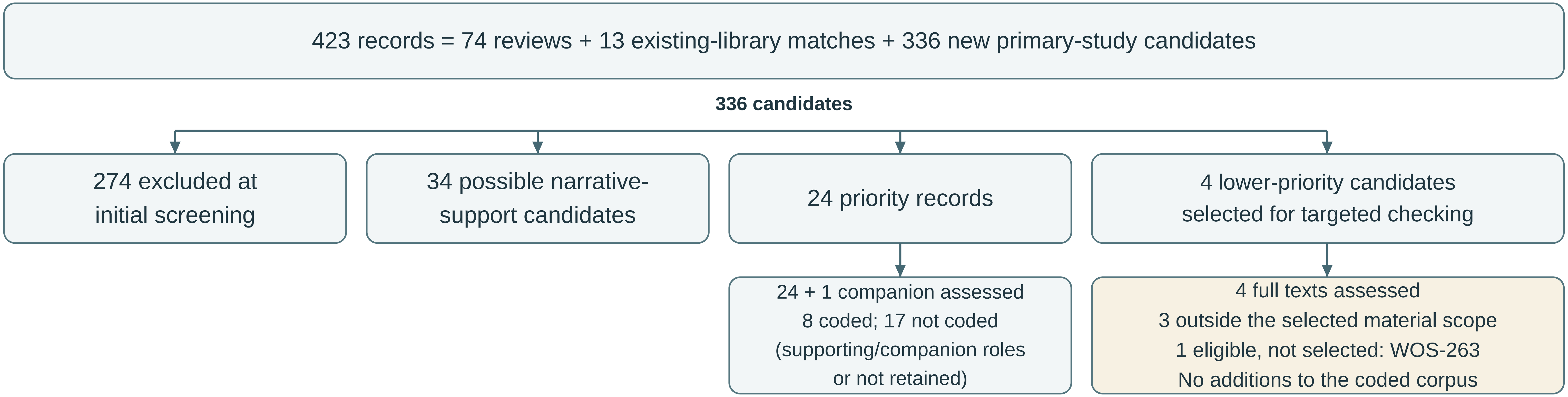

Counts are route-specific; repeated appearances across routes are not independent publications.

The 13 WoS matches refer to the existing library, not all overlap with Scopus exports.

Review searches were assessed separately: Scopus Q1 (2 recorded results), Q2 (142), WoS W4 (74).

Broad topical eligibility did not guarantee selection. This is not an exhaustive systematic-review flow.
